# Enhancing the NMR signals of plant oil components using hyperpolarisation relayed *via* proton exchange†

**DOI:** 10.1039/d3sc03078d

**Published:** 2023-08-29

**Authors:** Adel Alshehri, Ben. J. Tickner, Wissam Iali, Simon B. Duckett

**Affiliations:** a Department of Chemistry, Centre for Hyperpolarisation in Magnetic Resonance, University of York Heslington YO10 5NY UK simon.duckett@york.ac.uk

## Abstract

In this work, the limited sensitivity of magnetic resonance is addressed by using the hyperpolarisation method relayed signal amplification by reversible exchange (SABRE-Relay) to transfer latent magnetism from *para*-hydrogen, a readily isolated spin isomer of hydrogen gas, to components of key plant oils such as citronellol, geraniol, and nerol. This is achieved *via* relayed polarisation transfer in which an [Ir(H)_2_(IMes)(NH_2_R)_3_]Cl type complex produces hyperpolarised NH_2_R free in solution, before labile proton exchange between the hyperpolarisation carrier (NH_2_R) and the OH-containing plant oil component generates enhanced NMR signals for the latter. Consequently, up to *ca.* 200-fold ^1^H (0.65% ^1^H polarisation) and 800-fold ^13^C NMR signal enhancements (0.65% ^13^C polarisation) are recorded for these essential oils in seconds. Remarkably, the resulting NMR signals are not only diagnostic, but prove to propagate over large spin systems *via* a suitable coupling network. A route to optimise the enhancement process by varying the identity of the carrier NH_2_R, and its concentration is demonstrated. In order to prove utility, these pilot measurements are extended to study a much wider range of plant-derived molecules including rhodinol, verbenol, (1R)-*endo*-(+)-fenchyl alcohol, (−)-carveol, and linalool. Further measurements are then described which demonstrate citronellol and geraniol can be detected in an off-the-shelf healthcare product rose geranium oil at concentrations of just a few tens of μM in single scan ^1^H NMR measurements, which are not visible in comparable thermally polarised NMR experiments. This work therefore presents a significant expansion of the types of molecules amenable to hyperpolarisation using *para*-hydrogen and illustrates a real-world application in the diagnostic detection of low concentration analytes in mixtures.

## Abstract 2

The hyperpolarisation method SABRE-Relay can boost NMR signals of plant oil components by relayed proton exchange from a *para*-hydrogen enhanced carrier molecule. This allows low concentration analytes in natural oils to be detected more easily.

## Graphical abstract

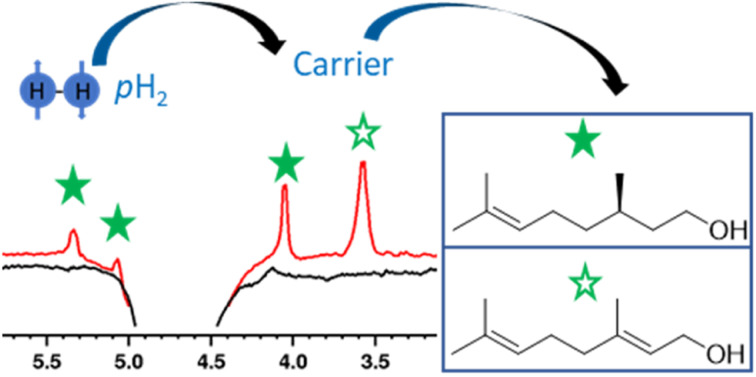

## Introduction

Molecular analysis and characterisation is the focus of significant attention by both academia and industry. Nuclear magnetic resonance (NMR) is often the method-of-choice in many instances as it can provide information about molecular structure without destruction of the sample. However, a limitation of magnetic resonance is its parts per million sensitivity. For example, in a typical 9.4 T magnet, only 1 in every 32 000 ^1^H spins are positively detected. Common ways to circumvent these sensitivity concerns are to use highly concentrated samples, or to perform signal averaging by making repeated measurements, and accept the associated time penalty of doing this.

Fortunately, in the last few decades, hyperpolarisation methods have emerged that can produce non-Boltzmann populations within the nuclear spin energy levels.^1,2^ A consequence of this is that molecules existing in this transient ‘hyper’-polarised state can be detected with NMR signals that can sometimes be many orders of magnitude larger than those recorded using thermally polarised NMR.^3–6^ The advantage of this approach is that molecules can then be detected at much lower concentrations (*i.e.* nM or μM rather than mM),^7–9^ or with reduced experiment time (seconds rather than tens of minutes or hours) which facilitates detection of transient species.^10–16^

In this work, the hyperpolarisation method relayed signal amplification by reversible exchange (SABRE-Relay) is employed which uses *para*-hydrogen (*p*-H_2_) gas as its source of enhanced NMR signals.^17,18^ The *p*-H_2_ feedstock is a spin isomer of hydrogen gas which is cheap and straightforward to produce.^19^ SABRE involves interaction of *p*-H_2_ and a to-be-hyperpolarised molecule with a polarisation transfer catalyst.^20–22^ Oxidative addition of *p*-H_2_ forms a hyperpolarised transition metal dihydride complex, with the hydrides giving dramatically enhanced NMR signals. Magnetisation can spread spontaneously from these hydride ligands to other ligands within the metal complex, which upon dissociation can give hyperpolarised NMR signals for the free ligand if dissociation occurs before the enhanced magnetisation has relaxed back to its thermal state.^23^ Hence, SABRE can produce hyperpolarised molecules in a continuous fashion as once the enhanced NMR signals have relaxed the substrate can rebind to the metal catalyst and receive fresh *p*-H_2_-derived polarisation.^24–31^

**Fig. 1:**
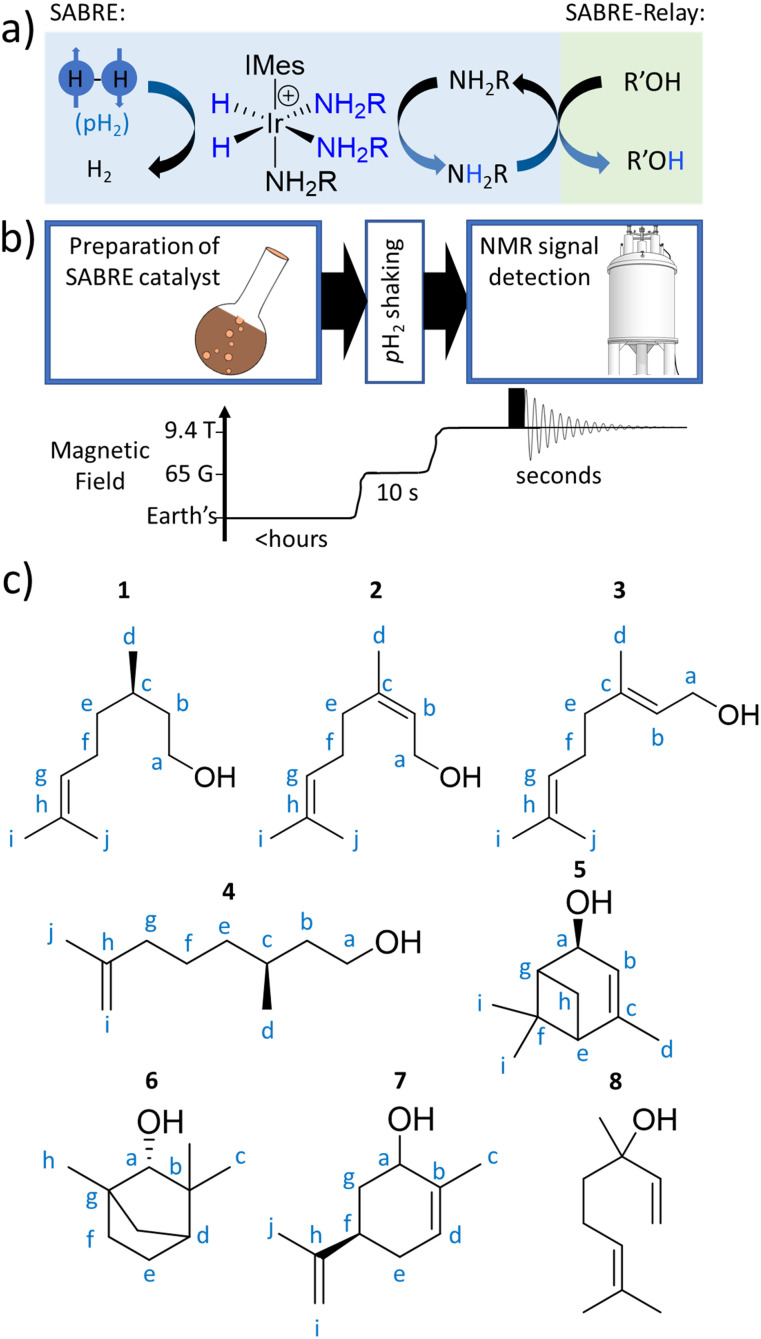
(a) SABRE can generate enhanced NMR signals for molecules in reversible exchange with a metal complex, with the metal catalyst also in simultaneous exchange with *p*-H_2_. The latent magnetism of *p*-H_2_ is unlocked by a symmetry-breaking pairwise oxidative addition reaction to form a metal dihydride. *p*-H_2_-derived spin order is then transferred through *J*-couplings to a ligated molecule. Subsequent dissociation allows enhanced NMR signals for a ligand, now free in solution, to be recorded. This ligand can act as a hyperpolarisation carrier and relay spin order to non-ligating molecules *via* proton exchange (SABRE-Relay). Note that polarisation can then propagate from the OH proton to other ^1^H or X nuclei sites in the secondary molecule. (b) Depiction of the hyperpolarisation process with associated time and magnetic field profiles. Upon formation of a magnetisation transfer catalyst *in situ*, the sample is manually shaken with *p*-H_2_ at 6.5 mT for 10 seconds, before being rapidly inserted into a 9.4 T spectrometer and spectral acquisition commenced. (c) The plant oil substrates 1–8 used in this work.

Traditional SABRE can sensitise molecules that readily ligate to the metal catalyst (including nitrogen heterocycles,^23,32–37^ nitriles,^38,39^ diazirines,^40^ keto-acids,^41,42^ amines^43^), but this scope can be extended to non-coordinating substrates *via* a relayed hyperpolarisation approach.^44,45^ In SABRE-Relay, substrates receive polarisation *via* proton exchange from a hyperpolarisation carrier (Fig. 1a), which itself can bind to the SABRE catalyst.^46^ The great benefit of this approach is that the NMR signals of a huge variety of motifs (alcohols,^47^ silanols,^48^ sugars,^49^ lactate esters^50^) can all be enhanced without any direct interaction with the SABRE catalyst. SABRE-Relay requires a non-coordinating aprotic solvent, and is typically performed in dichloromethane-*d*_2_ which allows for both catalyst and *p*-H_2_ solubility. A key consideration is that the solvent does not contain exchangeable protons that could interfere with the exchange of hyperpolarised proton from carrier to substrate. To this end, anhydrous solvents are beneficial as water, which provides an unwanted source of exchangeable protons, should be excluded.^47^

There are still a number of important questions that remain to be answered concerning the viability of SABRE-Relay as an analytical tool. These include assessing how SABRE-Relay performs with complex molecular scaffolds, if its responses are diagnostic for such materials, if whole spin-systems can become hyperpolarised or just particular sites, components, and if real-world samples can be targeted.

Here, we set out to answer these points by reference to the analysis of the important C_10_-based natural plant oils citronellol (1), nerol (2), and geraniol (3), which feature a common hydroxyl group (Fig. 1c). 1 is an acyclic monoterpenoid and a main component of many plant oils such as basil, citrus, citronella and lemongrass.^51,52^2 and 3 are molecules of very similar structure, but contain an additional unsaturated group within the carbon backbone (Fig. 1c). 2 and 3 are geometric isomers differing according to the arrangement of the alkene double bond (*E* or *Z*), and therefore the two cannot be distinguished by their mass alone. 2 is found in the oils of lemon grass and hops, and is often accompanied by 3 which itself is the major component of many oils such as rose oil, citronella oil, and palmarosa oil. 1–3 are colourless liquids with a rose-like scent and have similar properties (antibacterial, insect repellent) and uses (cosmetics, perfumes).^51,53^ The exchangeable OH proton should make these, and other plant oil components such as rhodinol (4), verbenol (5), (1R)-*endo*-(+)-fenchyl alcohol (6), (−)-carveol (7), and linalool (8), which are all commonly used in fragrances and detergents, amenable to SABRE-Relay. In this paper we therefore target real word complex analytes rather than small linear alcohols with limited functionality and complexity as currently reported.^46,47,54,55^ Boosting the NMR signals of these analytes will aid their detection and may have applications in mixture analysis and quality control in many off-the-shelf products.

## Results and discussion

The structures of the C_10_ plant oil substrates 1–8 studied in this work are shown in Fig. 1c. ^1^H and ^13^C NMR data for these starting materials is detailed in the ESI (Section S1†) for comparison purposes. Each are hyperpolarised using SABRE-Relay after synthesis of [Ir(H)_2_(IMes)(NH_3_)_3_]Cl *in situ*, by reaction of [IrCl(COD)(IMes)] (where COD = *cis*,*cis*-1,5-cyclooctadiene and IMes = 1,3-bis(2,4,6-trimethyl-phenyl)imidazole-2-ylidene) (5 mM) and NH_3_ (25–50 mM) with H_2_ (3 bar) in dichloromethane-*d*_2_ (0.6 mL) at room temperature for a few hours. [Ir(H)_2_(IMes)(NH_3_)_3_]Cl transfers the spin order of the *p*-H_2_ feedstock to the NH_2_R ligand upon shaking with *p*-H_2_ at a 6.5 mT polarisation transfer field (which is typically achieved in the stray field of our 9.4 T spectrometer).^43^ A magnetic field of 6.5 mT is selected as it has been reported to be optimal for polarisation transfer from *p*-H_2_-derived hydride ligands to ^1^H sites in ligated molecules.^56^ Samples were shaken for 10 seconds as this proved sufficient to allow catalytic build-up of hyperpolarisation, before signals begin to relax, and it yielded the largest NMR signal enhancements. The hyperpolarisation carrier NH_3_ was selected as it can be hyperpolarised efficiently, and the finite polarisation level achieved on the carrier is an important determinant in achieving high relayed NMR signal gains to secondary molecules.^47,50^ The initial metal : carrier : substrate ratio used was roughly 1 : 5 : 5 as previous studies have shown that this metal : carrier ratio gives an optimum SABRE response for the carrier molecule,^23,32,34^ and the 1 : 1 carrier : substrate ratio gives most efficient relayed transfer.^47,50^

### Enhancing ^1^H and ^13^C NMR signals for citronellol (1)

**Fig. 2:**
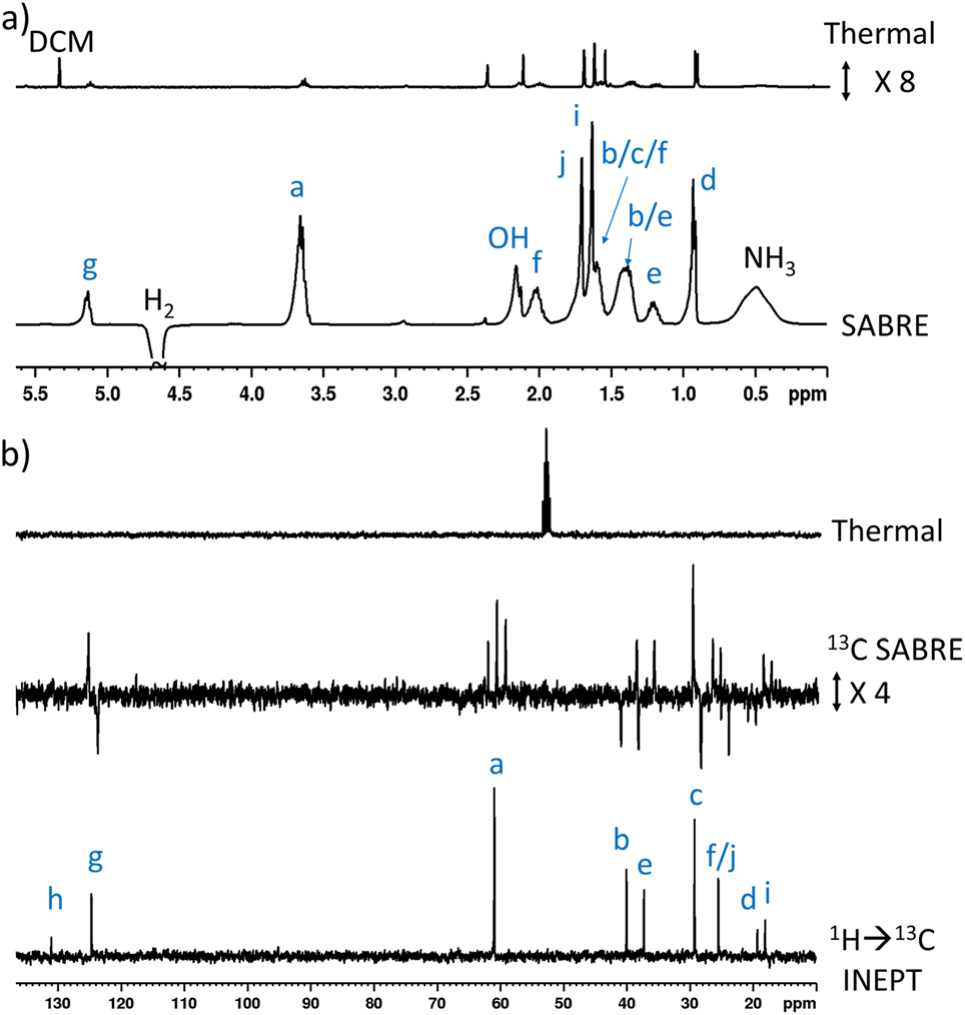
(a) Single scan thermally polarised (above) and SABRE-Relay hyperpolarised (lower) ^1^H NMR spectra of a sample of [IrCl(COD)(IMes)] (5 mM), NH_3_ (8 equiv.), citronellol (5 equiv.) and *p*-H_2_ (3 bar) in DCM-*d*_2_ (0.6 mL). The hyperpolarised spectrum is recorded immediately after shaking the sample for 10 seconds with fresh *p*-H_2_ at 6.5 mT. (b) Single scan thermally polarised (above) and SABRE-Relay hyperpolarised (middle and lower) ^13^C NMR spectrum of the same sample. The middle spectrum used a single 90° pulse for ^13^C detection whereas the INEPT sequence was used in the lower spectrum to transfer magnetisation from the ^1^H domain to the ^13^C domain *via* radiofrequency excitation. Note that the lower spectrum is not shown on the same vertical scale as the middle and upper spectra. The associated NMR signal enhancements are given in Table S4.†

When 1 (25 mM) and preformed [Ir(H)_2_(IMes)(NH_3_)_3_]Cl are shaken with *p*-H_2_ at 6.5 mT for 10 seconds, the hydride ligand signal for this species, alongside those of the bound and free NH_3_ ligands, exhibit hyperpolarisation due to SABRE.^43,46^ Additionally, all of the nine distinct CH proton environments in citronellol are also enhanced, due to SABRE-Relay. There was no evidence for direct interaction of 1 with the iridium SABRE catalyst (*i.e.* only the hydride for [Ir(H)_2_(IMes)(NH_3_)_3_]Cl is visible) which supports a relayed magnetisation transfer effect *via* proton exchange. Consequently, as anticipated, the highest signal gain (208-fold) was recorded for the CH_2_ protons closest to the exchanging OH group (Fig. 2a, see Table S4† for the signal enhancements for each site). These data confirm that the hyperpolarisation initially located in the OH proton readily cascades through the whole spin system of 1, albeit with generally lower signal enhancements at the sites more remote from the exchanging OH group. Despite this attenuation, ^1^H NMR signal enhancements are still observed for the terminal CH_3_ groups, at 61-fold and 63-fold (Fig. 2a and Table S4†).

SABRE-Relay is most efficient when performed at polarisation transfer fields of 6.5 mT as the process is ^1^H-driven and this field is optimum for achieving the maximum starting polarisation of the carrier available for relayed transfer.^47–50,56^ Heteronuclear NMR signals enhancements can also be achieved either *via* spontaneous ^1^H → X nuclei transfer at the 6.5 mT polarisation transfer field,^47,48,50,57–61^ or *via* radiofrequency-driven transfer at the spectrometer field.^27,62,63^ When the hyperpolarisation process was repeated, followed by single scan ^13^C NMR detection without ^1^H decoupling, enhanced ^13^C NMR signals for 1 were observed with antiphase characteristics, reflective of the fact that transfer is *via*^1^H. As expected, the largest signal enhancement is for the CH_2_ adjacent to the OH entry point (202-fold) (Fig. 2b and Table S4†). When ^1^H → ^13^C INEPT sequences are used after the *p*-H_2_ shaking process at 6.5 mT to transfer the ^1^H-derived magnetisation into the ^13^C domain all ten ^13^C responses can be detected in a single scan measurement (Fig. 2b). These decoupled spectra provide much clearer NMR signals as each ^13^C multiplet gives rise to just one peak, with no antiphase character. Hence, the complex spin system of 1 can be fully characterised at a 26 mM concentration using either ^1^H or ^13^C NMR measurements that take just a few seconds to record.

### Enhancing ^1^H and ^13^C NMR signals for nerol (2) and geraniol (3)

**Fig. 3:**
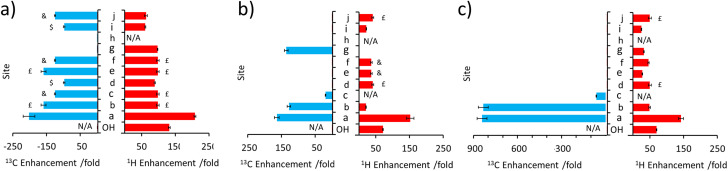
Comparison of ^1^H and ^13^C NMR signal enhancements for (a) 1 (b) 2 and (c) 3 when a sample of each (25 mM) is shaken with [IrCl(COD)(IMes)] (5 mM), NH_3_ (25 mM) and *p*-H_2_ (3 bar) in DCM-*d*_2_ (0.6 mL) for 10 seconds at 6.5 mT. Note that the symbols £, $ and & indicate signal overlap and therefore the signal enhancement quoted reflects an average of the two sites.

This approach was then extended to study 2 and 3 by shaking samples containing [IrCl(COD)(IMes)] (5 mM), NH_3_ (25 mM) and 2 or 3 (25 mM) with 3 bar *p*-H_2_ at 6.5 mT. In the case of 2, its ^1^H NMR signals proved to be enhanced in intensity compared to those derived from the Boltzmann condition (ESI, Fig. S4†). In this case, the signal for the CH_2_ group adjacent to the OH at *δ* 4.11 was 152-fold enhanced, per proton, while the olefinic proton of the adjacent double bond was enhanced by 21-fold (ESI, Table S5†). While polarisation was therefore able to cascade across the double bond, it resulted in small signal enhancements (20–45-fold) for all the remaining ^1^H sites in 2. The corresponding ^13^C SABRE experiments showed the largest signal enhancement for the *C*H_2_ site (164-fold), with discernible enhanced resonances for three other ^13^C sites (C_b_, C_c_, C_g_ according to the labelling in Fig. 1c). Notably, signal enhancements of 20-fold and 136-fold for C_c_ and C_g_ are seen respectively that confirm polarisation can spread across the double bond to remote sites (Fig. 3 and ESI, Fig. S5†), but as the level of proton signal gain for the remote sites is poorer than that for 1 the remaining ^13^C signals are not detected in these single scan measurements.

When 3 is examined, which is simply the *trans* isomer of 2, the ^1^H NMR signal enhancements appear comparable to those of 2 (Fig. 3). However, ^13^C polarisation now appears unable to spread across the alkene group. Consequently, enhanced ^13^C NMR signals for only the CH_2_ and CH sites are observed (Fig. 3 and ESI, Fig. S7†). One direct result of this is that the visible ^13^C NMR signal enhancements, which are derived from the ^1^H domain, are significantly higher in 3 (*ca.* 840-fold) when compared to those of 2 (*ca.* 100-fold) (Fig. 3) as the ^13^C polarisation is localised on fewer ^13^C sites. When comparing 1–3, the introduction of the unsaturated bond in 2 and 3 generally serves to limit polarisation transfer to sites remote from the OH group, with the effect being much more pronounced for the *trans* arrangement of the double bond in 3. This is reflective of a limitation if one aims to hyperpolarise a site remote from the OH group. However, this could also be exploited as a benefit as it serves to limit wastage of hyperpolarisation to remote sites, allowing it to be localised on a particular site which may yield, as in this case, chemically-discerning enhanced NMR signals. Other functionalities, such as ester groups, have also been shown to act as blocks to hyperpolarisation propagation as polarisation fails to efficiently transfer across this group spontaneously at this transfer field.^50–53^

### Extension to other substrates: rhodinol (4), verbenol (5), (1R)-*endo*-(+)-fenchyl alcohol (6), (−)-carveol (7), and linalool (8)

**Fig. 4:**
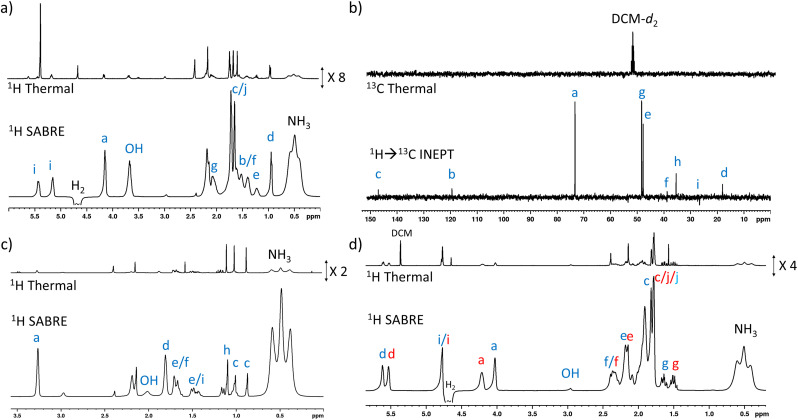
(a) Example single scan thermally polarised (above) and ^1^H SABRE-Relay hyperpolarised (lower) ^1^H NMR spectra for a sample of [IrCl(COD)(IMes)] (5 mM), NH_3_ (55 mM), 4 (25 mM) and *p*-H_2_ (3 bar) in DCM-*d*_2_ (0.6 mL). The associated NMR signal enhancements are given in the ESI, Table S7.† (b) Example single scan thermally polarised (above) and SABRE-Relay hyperpolarised ^1^H → ^13^C INEPT (lower) spectra for a sample of [IrCl(COD)(IMes)] (5 mM), NH_3_ (40 mM), 5 (25 mM) and *p*-H_2_ (3 bar) in DCM-*d*_2_ (0.6 mL). The associated signal enhancements are given in the ESI, Table S8.† (c) Example single scan thermally polarised (above) and ^1^H SABRE-Relay hyperpolarised (lower) ^1^H NMR spectra for a sample of [IrCl(COD)(IMes)] (5 mM), NH_3_ (40 mM), 6 (25 mM) and *p*-H_2_ (3 bar) in DCM-*d*_2_ (0.6 mL). The associated signal enhancements are given in the ESI, Table S9.† (d) Example single scan thermally polarised (above) and ^1^H SABRE-Relay hyperpolarised (lower) ^1^H NMR spectra for a sample of [IrCl(COD)(IMes)] (5 mM), NH_3_ (30 mM), 7 (25 mM) and *p*-H_2_ (3 bar) in DCM-*d*_2_ (0.6 mL). The associated signal enhancements are given in Table S10.† The resonance labels in red and blue correspond to the two diastereomers of 7. All hyperpolarised NMR spectra are recorded immediately after shaking the sample for 10 seconds with fresh *p*-H_2_ at 6.5 mT. Note that the hyperpolarised NMR spectra, and their thermally polarised counterparts, are shown on the same vertical scale, except for those in (b).

The complexity of the plant oil substrates was expanded further by testing several additional molecules including those with cyclic and bridged functionality. Namely, the hyperpolarisation of 4–8 was also achieved (Fig. 4) by shaking solutions containing them (25 mM), [IrCl(COD)(IMes)] (5 mM) and NH_3_ (25–60 mM) with 3 bar *p*-H_2_ in DCM-*d*_2_ (0.6 mL). In each case, ^1^H and ^13^C signal enhancements for all these substrates are observed (see ESI, Sections S2.4–S2.8†). In the case of 4, diagnostic ^1^H NMR signal enhancements for all sites are observed, with the largest (148-fold) being recorded for the C*H*_2_ site adjacent to the OH (Fig. 4a). Hyperpolarisation was also successfully transferred to ^13^C sites remote from OH, with the ^13^C NMR signal enhancements for the alkene *C*H site, attaining comparable signal enhancements (168-fold for *C*H compared to 135-fold for the *C*H_2_OH site). In order to rationalise this behaviour it should be noted that like 1, 4 possess a ^3^*J*_HH_^1^H–^1^H coupling network that spans the majority of the molecule.

Substrates 5, 6, and 7 have a different type of molecular scaffold as they are cyclic OH-containing molecules, and in the case of 5 and 6 contain bridged rings. These substrates are all secondary alcohols, which reflects a difference from the linear form of the primary alcohols described so far. Nonetheless, these substrates proved to hyperpolarise using SABRE-Relay. In the case of 5, ^1^H NMR signal enhancements of 302-fold were recorded for the proton adjacent to the OH, and high signal gains (*ca.* 100–200-fold) were observed for all the other ^1^H sites, with the exception of the weaker 38-fold enhancement seen for the alkene proton that connects to the CHOH group *via* a small ^3^*J*_HH_ coupling. ^13^C NMR enhancements were also observed (Fig. 4b) for all sites other than C_d_ and C_i_, which are each attached to quaternary carbons. In this case the enhancement for the site closest to the OH (164-fold) was not as large as those of the other sites; for example, the two bridgehead carbons (281-fold) or the quaternary centre (205-fold).

Interestingly, 6, which is a structural isomer of 5, can be hyperpolarised using SABRE-Relay, but with a much lower efficiency. For example, the largest ^1^H NMR signal gain was also recorded for the proton adjacent to the OH, but it was now just 45-fold (Fig. 4c). No ^13^C NMR enhancements were now observed. These differences in performance are hypothesised to result from the spin topology of the substrate. In the case of 6, there are now two quaternary centres immediately adjacent to the CHOH group. These could serve to limit polarisation transfer through the substrate as there are no ^3^*J*_HH_ couplings from the R_2_C*H*OH proton for propagation, which now must occur through smaller ^4^*J*_HH_ couplings if the two alkyl arms of R_2_C*H*OH are to become hyperpolarised. Hence, a slower labile proton exchange rate may therefore be beneficial for polarisation transfer to these sites.

7 is a non-fused cyclic secondary alcohol, and it too can be hyperpolarised using SABRE-Relay. Interestingly, 7 existed as a mixture of two diastereomers with distinctive ^1^H and ^13^C NMR resonances for each. This was particularly evident for the sites closest to the chiral CR_2_HOH group, which differ in the two diastereomers. For example, a *ca.* 0.2 ppm and *ca.* 2.5 ppm difference in the ^1^H and ^13^C NMR resonances of the diastereomeric *C*R_2_*H*OH sites are observed (Fig. 4d). This allows these two diastereomers to be easily distinguished based on these characteristic resonances. All the ^1^H sites for both diastereomeers of 7 were enhanced by SABRE-Relay, with the largest enhancements being recorded for the CR_2_*H*OH proton (*ca.* 70-fold) (ESI, Table S10†). In ^1^H SABRE-Relay measurements, the NMR signal enhancements were the same for the same site in each diastereomer which, taken with the fact these two isomers are present in a 1 : 1 ratio, suggests that factors such as OH exchange, propagation of polarisation within the substrate, and relaxation, are the same for each diastereomer. Therefore, as each diastereomer is hyperpolarised to the same extent using SABRE-Relay, the method could be used to reliably quantify the ratio of diastereomers from single scan hyperpolarised spectra in this case. To make such distinctions using hyperpolarised ^13^C SABRE-Relay experiments is much more challenging due to peak multiplicity and signal overlap.

Finally, hyperpolarisation of the tertiary alcohol 8 can be achieved, albeit it with much lower efficiency. For example, the largest ^1^H NMR signal enhancement recorded was just 65-fold (ESI, Table S11 and Fig. S16†), and no ^13^C hyperpolarisation was observed, even when ^1^H → ^13^C INEPT experiments were used. This reflects the fact that tertiary alcohols contain no ^3^*J*_Hx–OH_ coupling as there is no proton directly bound to the alcohol carbon. Therefore, propagation of polarisation *via J* coupling must rely on a less-efficient ^4^*J*_Hx–OH_ coupling. The lower efficiency of polarisation propagation in tertiary alcohols compared to secondary and primary alcohols has been noted before.^47^

### Optimising and understanding SABRE-Relay by varying carrier and concentrations

Previous studies have highlighted that the starting polarisation of the carrier NH proton is an important determinant in the resultant level of polarisation relayed on to secondary molecules.^43,47,50^ Therefore, the efficiency of polarisation transfer from the metal dihydride SABRE catalyst to the carrier NH in a traditional SABRE process will be closely linked to the effectiveness of SABRE-Relay hyperpolarisation of a secondary molecule. Accordingly, the identity of the carrier NH (and its hyperpolarisation efficiency) is one of the most important factors in determining the magnitude of relayed NMR signal enhancements for OH-containing molecules. To this end, experiments were performed using 1 and a series of other NH carrier molecules at the same metal : carrier : substrate ratios to determine the effect of carrier identity on the magnitude of ^1^H NMR enhancements of 1.

The carrier phenethylamine was examined and it yielded ^1^H NMR signal enhancements for 1 significantly lower than those attained using NH_3_ (maximum of 6-fold compared to 208-fold for the CH_2_ proton next to the OH, see ESI Fig. S17 and Table S12†). When the experiment was repeated using phenethylamine-*d*_4_, ^1^H NMR signal enhancements for 1 were *ca.* 4–7 times higher (ESI Table S12†) which reflects the fact that deuteration of the carrier molecule can serve to reduce polarisation wastage within the amine. By localising polarisation on the NH, it increases the amount available for relayed transfer and these effects have been observed previously for other indirectly polarised molecules.^47–49^ The carrier benzylamine-*d*_7_ gave signal enhancements comparable to those achieved using phenethylamine-*d*_4_ (ESI, Table S12†). Similar trends of SABRE-Relay efficiency in the order of NH_3_ > benzylamine-d_7_ > phenthylamine-*d*_4_ > phenethylamine; and NH_3_ ∼ benzylamine-*d*_7_ > phenthylamine-*d*_4_ are observed for 2 and 3 respectively (ESI Tables S13 and S14†). These findings are consistent with previous observations that NH_3_ can be hyperpolarised by SABRE more efficiently than both phenethylamine and benzylamine.^43^

**Fig. 5:**
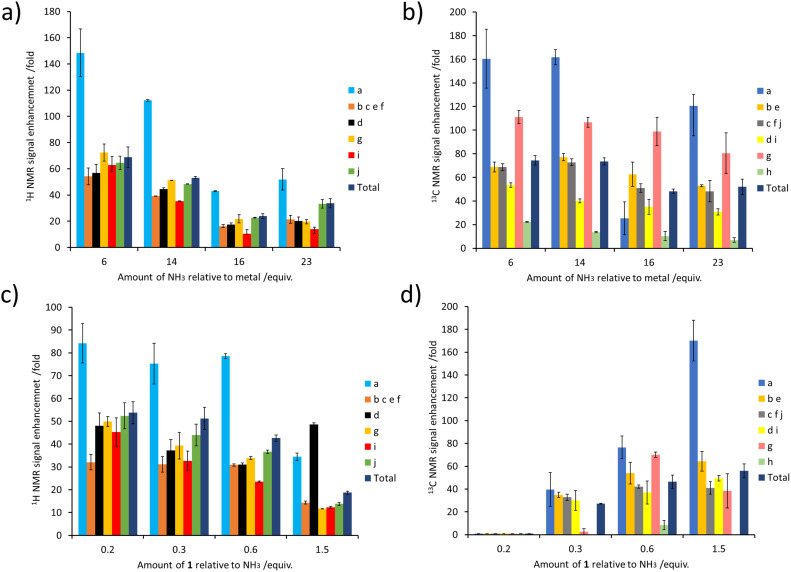
(a and b) Effect of varying the NH_3_ concentration on the NMR signal enhancements of 1. Samples of 1 (60 mM) were shaken with [IrCl(COD)(IMes)] (5 mM), NH_3_ (indicated amount) and *p*-H_2_ (3 bar) in DCM-*d*_2_ (0.6 mL) for 10 seconds at 6.5 mT. (c and d) Effect of varying the concentration of 1 on its NMR signal enhancements. Samples of 1 (indicated amount) were shaken with [IrCl(COD)(IMes)] (5 mM), NH_3_ (60 mM) and *p*-H_2_ (3 bar) in DCM-*d*_2_ (0.6 mL) for 10 seconds at 6.5 mT.

The concentration of NH_3_ can also have a large effect on SABRE-Relay efficiency. This was demonstrated by preparing samples containing IrCl(COD)(IMes) (5 mM), 1 (60 mM) and varying but precise amounts of NH_3_ in dichloromethane-*d*_2_ (0.6 mL). After forming the active magnetisation transfer catalyst [Ir(H)_2_(IMes)(NH_3_)_3_]Cl, the samples were shaken with *p*-H_2_ for 10 seconds and hyperpolarised NMR spectra recorded. These NMR spectra revealed that as the amount of NH_3_ relative to the metal centre increased from 30 to 115 mM there is a general decrease in the level of ^1^H signal enhancement seen for 1 (for individual sites as well as the total ^1^H polarisation level) (Fig. 5a). The same trend was seen for the corresponding ^13^C NMR signal enhancements (Fig. 5b). Similar trends are observed when the NH_3_ concentration was raised from 25 to 60 mM in samples containing a much lower citronellol loading (30 mM compared to 60 mM, see ESI, Fig. S18†). This reflects optimal carrier/metal exchange rates at metal : carrier ratios of 1: *ca.* 5–10.

Samples were also tested where the concentration of NH_3_ was fixed at 60 mM, and that of 1 was varied. These experiments resulted in a drop in ^1^H NMR signal enhancements for 1 as its ratio relative to NH_3_ increased (Fig. 5c). The essentially linear behaviour seen would be consistent with a chemical process involving an inverse first order dependence on the concentration of the corresponding reagent that is being changed. Therefore, the ^1^H NMR signal enhancement of 1 appears inversely proportional to the excess of 1 relative to NH_3_. Additionally, these results indicate that a smaller sub stoichiometric excess of 1 relative to NH_3_ can improve the level of enhancement, which could allow easier detection of its ^1^H NMR resonances even if they are present in a very small quantity. Previous studies on simple alcohol substrates like methanol were reported to give an optimum signal enhancement when the alcohol and carrier ratio was *ca.* 1 : 1 and this difference reflects the fact that for each substrate the proton exchange rate and relaxation times will be different. Consequently, we deduce that the optimum exchange rate for the most efficient polarisation transfer, and hence sensitisation, may be produced for different carrier : substrate ratios depending on the specific substrate investigated.

Surprisingly, the corresponding ^13^C NMR signal enhancement trend proved opposite, with an increase observed as the amount of 1 increased (Fig. 5d). Although ^13^C NMR signal enhancements are closely linked to the size of the ^1^H magnetisation that they are derived from, and this ^1^H → ^13^C transfer has recently been reported to occur over short distances *via* cross relaxation,^50,64^ a spin diffusion mechanism may also be possible, particularly over longer distances. This highlights the tension between an optimum carrier : substrate ratio for both ^1^H polarisation and subsequent ^1^H → ^13^C transfer.

### Detection of 1 and 3 in rose geranium oil

We next turn our attention to using the enhanced ^1^H NMR resonances provided by SABRE-Relay to detect some of these plant oil components in mixtures. We therefore purchased rose geranium essential oil and examined if its components could be detected and analysed using NMR enhanced by SABRE-Relay. To this end, the complex [Ir(H)_2_(IMes)(NH_3_)_3_]Cl was prepared *in situ* in DCM-*d*_2_ (*vide supra*) and then an addition of rose geranium oil was made and the sample shaken with fresh *p*-H_2_ at 3 bar before immediate NMR analysis. According to the manufacturer, the major components of the examined rose geranium oil are 1 (21.1%), 3 (17.7%) and 8 (10.2%), with 2 also present, but only at 1%. ^1^H and ^13^C NMR spectra for rose geranium oil are shown in the ESI, Fig. S19 and S20.†

In an initial experiment, an addition of rose geranium oil was made to [Ir(H)_2_(IMes)(NH_3_)_3_]Cl to give concentrations of 1 (26.5 mM) and 3 (20.6 mM) comparable to those used when each was examined independently. When this sample was hyperpolarised using SABRE-Relay, enhanced ^1^H NMR resonances for both 1 and 3 were observed. There is a distinctive hyperpolarised resonance at *δ* 3.67 which corresponds to 1. There are also characteristic resonances at *δ* 5.43 and *δ* 4.13 attributed to 3. However, although the chemical shifts of 2 are similar to those of 3, and their ^1^H SABRE performance is similar, these resonances are assigned as 3, as 2 is present at just 1% of the rose geranium oil. Additional enhanced signals at *δ* 1.38, 1.19 and 0.94 are assigned as 1, although the region between 1 and 3 ppm suffers from spectral crowding due to overlap of resonances for components of the mixture, in addition to those of NH_3_ and the IMes ligands of the SABRE catalyst. This is one factor that prevents detection of analytes such as 7 (present at 10%) as sites that are most significantly hyperpolarised are in this crowded chemical shift window. These hyperpolarised ^1^H NMR signals are all weakly enhanced by up to 36-fold for 1 (the C*H*_2_OH site at *δ* 3.67), and for 25-fold for 3 (the C*H*_2_OH site at *δ* 5.43) (see ESI, Table S15†).

**Fig. 6:**
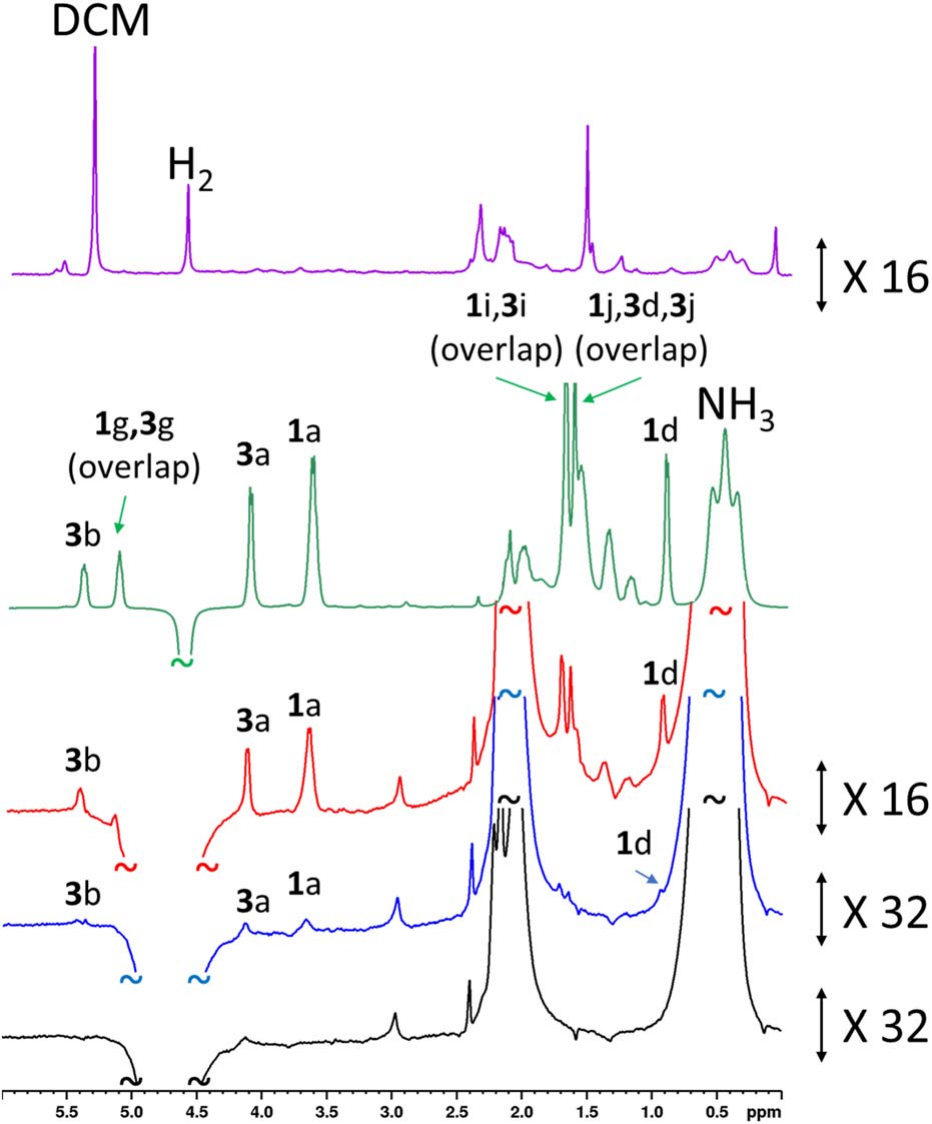
(a) SABRE-Relay hyperpolarised ^1^H NMR control experiment for [Ir(H)_2_(IMes)(NH_3_)_3_]Cl formed *in situ* (lower, black) with a spectrum for the same sample after addition of rose geranium oil (1: 37 μM and 3: 29 μM) (blue). NMR spectra recorded after the addition of more rose geranium oil (1: 92 μM and 3: 72 μM, red spectrum and 1: 554 μM and 3: 431 μM, green spectrum). A thermally polarised NMR spectrum is shown at the top (purple) for comparison (1: 92 μM and 3: 72 μM). The hyperpolarisation process involved shaking a sample for 10 seconds with fresh *p*-H_2_ at 6.5 mT. Resonances are labelled according to the scheme shown in Fig. 1c.

These experiments were repeated when the amount of rose geranium oil added was significantly lower to allow detection of sub-mM concentrations of 1 and 3 (Fig. 6). Three samples were prepared with 1 concentrations of 554 μM, 92 μM and 40 μM and accompanying 3 concentrations of 431 μM, 72 μM and 29 μM respectively. These concentrations were determined from ^1^H NMR by measuring the concentration of a stock solution of rose geranium oil using an internal standard. The distinctive enhanced ^1^H NMR resonances for both 1 (*δ* 3.67, 0.94) and 3 (*δ* 5.43, 4.13) are again readily observed in the most concentrated of these samples (554/431 μM). Enhanced signals at *δ* 5.15, 1.71 and 1.64 cannot be assigned as either 1 or 3 as both have signals with these chemical shifts. The ^1^H NMR signal enhancements are now much more significant compared to the sample containing analyte concentrations of *ca.* 21–27 mM, with 98-fold and 127-fold enhancements for the distinctive *δ* 3.65 and *δ* 5.43 resonances of 1 and 3 respectively (Fig. 6 and ESI Table S15†). Excitingly, these enhanced resonances can all be distinguished at much lower substrate concentrations of 92/72 μM, and 37/29 μM (Fig. 6). We expect that for even lower analyte concentrations, distinguishing the hyperpolarised signals from the baseline will become more challenging. Consequently, the detection limit for these oils under these conditions using single-scan SABRE-Relay hyperpolarised ^1^H NMR is estimated to be in the low tens of μM. Whilst the quantitative determination of unknown concentrations using SABRE hyperpolarised NMR in conjunction with a standard additional method has been reported,^7,29,65,66^ it is extremely challenging to apply that method to this example, as repeated analyte additions and multiple shaking can serve to lower the amount of NH_3_ in the sample (*i.e.* by placing the solution under vacuum repeatedly to remove the spent H_2_). Changing the amount of NH_3_ can have a strong effect on the NMR signal enhancements of the substrate and therefore the amount of the carrier must be kept constant throughout multiple *p*-H_2_ shakes. Nevertheless, we have shown that low (*ca.* a few tens of micromolar) concentrations of naturally occurring oils can be detected in just a single scan ^1^H NMR experiment. These concentrations of material cannot be readily observed in an equivalent single-scan thermally polarised NMR measurement and would require signal averaging to be detected.

## Experimental

All NMR measurements were carried out on a 400 MHz Bruker Avance III spectrometer at 298 K unless otherwise stated. *Para*-hydrogen (*p*-H_2_) was produced by passing hydrogen gas over a spin-exchange catalyst (Fe_2_O_3_) at 28 K and used for all hyperpolarisation experiments. This method produces constant *p*-H_2_ with *ca.* 99% purity. ^1^H (400 MHz) and ^13^C (100.6 MHz) NMR spectra were recorded with an internal deuterium lock. Chemical shifts are quoted as parts per million and referenced to residual CHDCl_2_. The DCM-*d*_2_ used throughout was dried by stirring with CaH_2_ for a few days at room temperature and stored using molecular sieves before the NMR samples were prepared in a glovebox as a precaution to limit moisture in the samples (water has exchangeable protons that can become hyperpolarised instead of the target substrate). ^13^C NMR spectra were recorded with broadband proton decoupling. Coupling constants (*J*) are quoted in Hertz. All starting compounds were purchased from Sigma Aldrich, Fluorochem, or Alfa-Aesar and used as supplied without further purification.

The shake & drop method was employed for recording hyperpolarised SABRE NMR spectra. Samples were prepared in a 5 mm NMR tube that was fitted with a J. Young's tap. The iridium precatalyst used was [IrCl(COD)(IMes)] (where IMes = 1,3-bis(2,4,6-trimethyl-phenyl)imidazole-2-ylidene and COD = *cis*,*cis*-1,5-cyclooctadiene) and was synthesised in our laboratory according to a literature procedure.^67^ The NMR samples were subsequently degassed by three freeze–pump–thaw cycles using a Schlenk line before filling the tube with *p*-H_2_ at 3 bar overpressure. Once filled with *p*-H_2_, the tubes were shaken vigorously for 10 seconds at *ca.* 6.5 mT. Immediately after that, the NMR tubes were put inside the spectrometer for immediate NMR detection.

NMR signal enhancements were calculated by dividing the hyperpolarised integral intensity by the corresponding intensity from a 1 scan thermal recorded and processed under the same conditions. Both hyperpolarised and thermally polarised spectra were recorded on the same sample using the same spectrometer settings. ^13^C NMR signal enhancements were calculated by reference to the thermally polarised solvent and are calculated according to the equation previously reported.^68^^1^H → ^13^C INEPT sequences used a mixing time corresponding to a 122 Hz ^1^H–^13^C coupling.^50^

## Conclusions

The successful hyperpolarisation of the complex plant-based natural products (essential oils) including citronellol, nerol, geraniol, rhodinol, verbenol, (1R)-*endo*-(+)-fenchyl alcohol, (−)-carveol, and linalool using SABRE-Relay is presented. This is achieved by relayed polarisation transfer *via* their hyperpolarised OH protons that are created by chemical exchange with hyperpolarised NH protons of the polarisation carrier (NH_3_, benzylamine-d_7_, phenethylamine, phenethylamine-*d*_4_), formed *via* SABRE. Strong NMR signal enhancements are seen for many of their ^1^H and ^13^C NMR signals which prove to be diagnostic of their identity.

The substrates citronellol, nerol and geraniol present enhancements which reach 200-fold for ^1^H and 800-fold for ^13^C (0.65% polarisation for both ^1^H and ^13^C) for the site closest to the OH group. The carrier ammonia proved to consistently out-perform the other amines tested, and the protons of the C*H*_2_OH group all receive the highest level of polarisation. Despite many of the examined substrates containing unsaturated functionality, no evidence for their hydrogenation by the SABRE catalyst was observed, which is attributed to preferential binding of the carrier. Thus, we prove it is possible to successfully reversibly sensitise OH-containing molecules that also have unsaturated functionality, without observing competing hydrogenative effects.

In the case of citronellol, rhodinol, and verbenol the spin systems can be traversed by a series of ^3^*J*_HH_ couplings until the remote alkene moiety is reached. This spin topology enables most of the ^1^H and ^13^C NMR signals in these molecules to receive polarisation. In contrast, nerol and geraniol show different behaviour as the former proved to exhibit weaker ^1^H NMR signal gains for all its resonances, whilst the later only showed gains for the MeC=CHCH_2_OH moiety. This is the result of the *trans* RC=CH spin–spin coupling enabling poor polarisation propagation across the double bond, thereby allowing very high signal enhancements to remain on the C=CHCH_2_OH moiety. Furthermore, when dealing with a tertiary alcohol like linalool there is no proton on the carbon bearing the OH and very poor transfer results. Hence, we demonstrate that whilst the spin topology of the target is important in controlling which sites receive polarisation, in all cases enhanced signals are seen for these materials.

These measurements confirm the benefit of using SABRE-Relay as a tool to examine OH-containing natural oils, as exemplified by the ready differentiation of diastereomers of (−)-carveol (which are indistinguishable by mass). Further examples of detecting citronellol and geraniol in off-the-shelf rose geranium oil using single scan hyperpolarised ^1^H NMR spectroscopy at concentrations as low as *ca.* 30 μM show how this technique can be applied to real-world molecular analysis. Furthermore, we have assessed realistic concentration limits and the potential interference of other analyte components in mixtures, particularly water which should be minimised where possible. Further optimisation of SABRE-Relay to give larger NMR signal enhancements will lower these detection limits and combination with 2D NMR or high field methods may prove useful to reduce peak overlap for the most complex of mixtures.^69–72^

## Data availability

The raw NMR data collected in this study is available from the University of York data repository (https://doi.org/10.15124/e632d8dd-3e74-4eaa-801c-43feb59c303e).

## Author contributions

AA – investigation, BJT – conceptualisation; investigation; writing – original draft, WI – conceptualisation; investigation (early-stage measurements), SBD – conceptualisation; writing – review and editing; supervision, funding acquisition.

## Conflicts of interest

There are no conflicts to declare.

## Supplementary Material

SC-014-D3SC03078D-s001
