# Supplementary material for: Enhancing the NMR signals of plant oil components using hyperpolarisation relayed via proton exchange
Source: Chem Sci. 2023 Aug 29;14(36):9843–53. doi: 10.1039/d3sc03078d (PMC10510812; doi:10.1039/d3sc03078d)
Supplement: SC-014-D3SC03078D-s001 [file SC-014-D3SC03078D-s001.pdf]

Electronic Supplementary Material (ESI) for Chemical Science.
This journal is © The Royal Society of Chemistry 2023

Supporting Information

Enhancing the NMR signals of plant oil components using hyperpolarisation relayed *via* proton exchange

Adel Alshehri, [a$] Ben. J. Tickner, [a] Wissam Iali [a,£] and Simon B. Duckett*[a]

[a.] *Centre for Hyperpolarisation in Magnetic Resonance, Department of Chemistry, University of York, Heslington, YO10 5NY, United Kingdom*
[$]*Current Address: Mr. A Alshehri, Department of Radiological Sciences, College of Applied Medical Sciences, Taif University, Taif 21944, Saudi Arabia*
[£]*Current Address: Dr. W. Iali, Department of Chemistry, King Fahd University of Petroleum and Minerals (KFUPM), Dhahran, 31261, Kingdom of Saudi Arabia.*
**Corresponding Author email: simon.duckett@york.ac.uk*

Table of Contents

S1: Characterisation of selected starting materials using NMR

S1.1 Citronellol

S1.2 Nerol

S1.3 Geraniol

S2: SABRE-Relay hyperpolarisation

S2.1 SABRE-Relay hyperpolarisation of **1**

S2.2 SABRE-Relay hyperpolarisation of **2**

S2.3 SABRE-Relay hyperpolarisation of **3**

S2.4 SABRE-Relay hyperpolarisation of **4**

S2.5 SABRE-Relay hyperpolarisation of **5**

S2.6 SABRE-Relay hyperpolarisation of **6**

S2.7 SABRE-Relay hyperpolarisation of **7**

S2.8 SABRE-Relay hyperpolarisation of **8**

S3: Optimising SABRE-Relay hyperpolarisation of **1**

S4.1 Effect of carrier on SABRE-Relay hyperpolarisation of **1**

S4.2 Effect of carrier on SABRE-Relay hyperpolarisation of **2**

S4.3 Effect of carrier on SABRE-Relay hyperpolarisation of **3**

S4.4 Effect of NH_3 concentration on SABRE-Relay hyperpolarisation of **1**

S4: Hyperpolarisation of rose geranium oil mixture

S4.1 Characterisation of rose geranium oil

S4.2 Hyperpolarisation experiments

S1: Characterisation of selected starting materials using NMR

S1.1: Citronellol

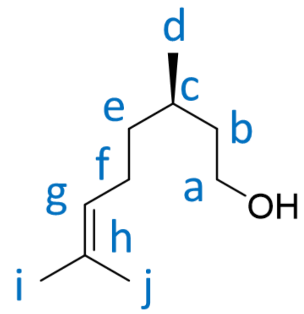

Figure S1: Structure of 1. The NMR data for the labelled resonances is given in Table S1.

Table S1: NMR characterisation data for 1 in dichloromethane-d_2 at 298 K. The resonance labels correspond to those shown in Figure S1.

Resonance	^{1}H (ppm)	^{13}C (ppm)	^{1}H T_1 (seconds)
OH	2.14 (1H)	-	
a	3.65 (*m*, 2H)	60.9 (*t* $^{1}J_{C9H9}$ = 139 Hz)	5.0
b	1.39 (*m*, 1H), 1.64 (*m*, 1H)	39.9 (*t* $^{1}J_{C4H4}$ = 124 Hz)	5.9
c	1.59 (*m*, 1H)	29.2 (*d* $^{1}J_{C3H3}$ = 125 Hz)	9.4
d	0.94 (*d* $^{3}J_{H1H3}$ = 6.6 Hz, 3H)	19.2 (*q* $^{1}J_{C1H1}$ = 123 Hz)	3.0
e	1.22 (*m*, 1H), 1.38 (*m*, 1H)	37.2 (*t* $^{1}J_{C2H2}$ = 126 Hz)	3.7
f	2.03 (*m*, 1H), 1.67 (*m*, 1H)	25.4 (*t* $^{1}J_{C7H7}$ = 126 Hz)	3.7
g	5.14 (*m*, 1H)	124.6 (*d* $^{1}J_{C10H10}$ = 149 Hz)	9.8
h	-	131.1 (*s*)	-
i	1.65 (*s*, 3H)	17.3 (*q* $^{1}J_{C5H5}$ = 119 Hz)	4.7
j	1.72 (*s*, 3H)	25.4 (*q* $^{1}J_{C6H6}$ = 123 Hz)	7.6

S1.2: Nerol

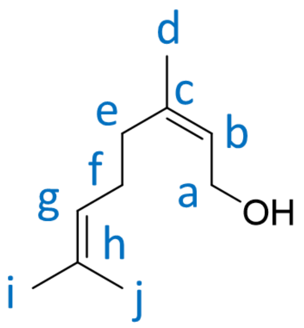

Figure S2: Structure of 2. The NMR data for the labelled resonances is given in Table S2.

Table S2: NMR characterisation data for 2 in dichloromethane-d_2 at 298 K. The resonance labels correspond to those shown in Figure S2.

Resonance	^{1}H (ppm)	^{13}C {^{1}H} (ppm)	^{1}H T_1 (seconds)
OH	1.57 (*br*, 1H)	-	
a	4.11 (*d*, 2H)	58.84	6.4
b	5.45 (*t*, 1H)	124.54	13.1
c	-	139.55	-
d	1.72 (*s*, 3H)	25.44	4.8
e	2.18 (*d*, 2H) (overlap)	31.91	3.9
f	2.18 (*d*, 2H) (overlap)	26.54	3.9
g	5.16 (*m*, 1H)	123.94	9.3
h	-	132.19	-
i	1.65 (*s*, 3H)	17.39	4.6
j	1.78 (*s*, 3H)	23.06	8.0

S1.3: Geraniol

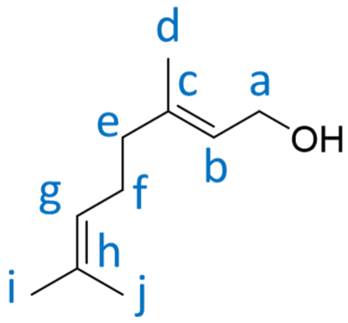

Figure S3: Structure of 3. The NMR data for the labelled resonances is given in Table S3.

Table S3: NMR characterisation data for 3 in dichloromethane-d_2 at 298 K. The resonance labels correspond to those shown in Figure S3.

Resonance	^{1}H (ppm)	^{13}C {^{1}H} (ppm)	^{1}H T_1 (seconds)
OH	1.57 (*br*, 1H)	-	
a	4.14 (*d*, 2H)	59.3	6.2
b	5.42 (*t*, 1H)	123.7	9.5
c	-	139.3	-
d	1.70 (*s*, 3H)	16.0	6.9
e	2.08 (*d*, 2H)	39.5	3.3
f	2.17 (*d*, 2H)	26.5	3.3
g	5.15 (*m*, 1H)	124.0	10.2
h	-	131.6	-
i	1.65 (*s*, 3H)	25.4	4.9
j	1.72 (*s*, 3H)	17.5	7.8

S2: SABRE-Relay hyperpolarisation

S2.1: SABRE-Relay hyperpolarisation of 1

A sample of **1** (25 mM) was exposed to $p\mathrm{H}_2$ (3 bar) in the presence of NH_3 (25 mM) and the precatalyst [IrCl(COD)IMes] for several hours. The sample was then shaken with $p\mathrm{H}_2$ at 6.5 mT for 10 seconds before spectral acquistion was performed at 9.4 T. Example ^{1}H and ^{13}C NMR spectra are shown in Figure 2 in the main paper, with the associated NMR signal enhancments being given in Table S4.

Table S4: ^{1}H and ^{13}C NMR signal enhancements for 1 measured from a sample containing [IrCl(COD)(IMes)] (5 mM), NH_3 (25 mM), 1 (25 mM) and $p\mathrm{H}_2$ (3 bar) in DCM-d_2 (0.6 mL). ^{13}C NMR signal enhancements are calculated from data recorded with a single 90° detection pulse.

^{1}H		^{13}C	
Resonance	Enhancement (per proton, fold)	Resonance	Enhancement (per ^{13}C, fold)
OH	132 ± 3	OH	-
a	208 ± 3	a	202 ± 17
b, c, e, f (overlap)	98 ± 4	b and e (overlap)	159 ± 7
d	89 ± 2	c, f, j (overlap)	125 ± 2
g	97 ± 1	d and i (overlap)	98 ± 2
i	61 ± 1		
j	63 ± 4		
Total	108 ± 4	Total	130 ± 2

S2.2: SABRE-Relay hyperpolarisation of 2

A sample of **2** (25 mM) was exposed to $p\mathrm{H}_2$ (3 bar) in the presence of NH_3 (25 mM) and the precatalyst [IrCl(COD)IMes] for several hours. The sample was then shaken with $p\mathrm{H}_2$ at 6.5 mT for 10 seconds before spectral acquistion was performed at 9.4 T. Example ^{1}H and ^{13}C NMR spectra are shown in Figures S4 and S5 respecitvely, with the associated NMR signal enhancments being given in Table S5.

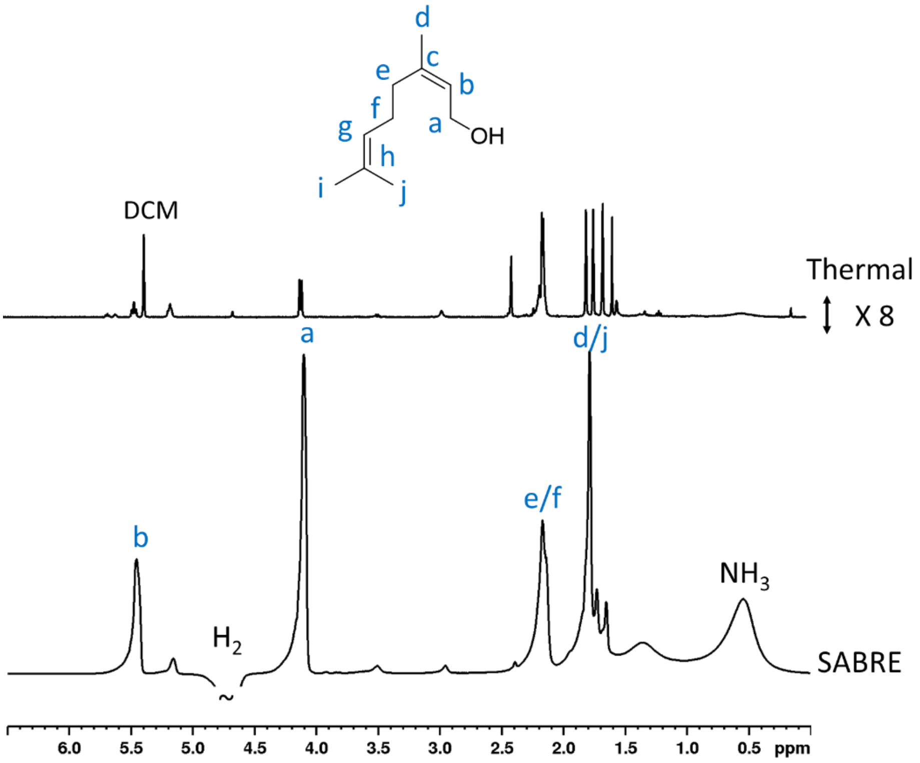

Figure S4: Single scan thermally polarised (above) and SABRE-Relay hyperpolarised (lower) ^{1}H NMR spectra for a sample of [IrCl(COD)(IMes)] (5 mM), NH_3 (25 mM), 2 (25 mM) and pH_2 (3 bar) in DCM-d_2 (0.6 mL). The hyperpolarised NMR spectrum is recorded immediately after shaking the sample for 10 seconds with fresh pH_2 at 6.5 mT. The associated signal enhancements are given in Table S5.

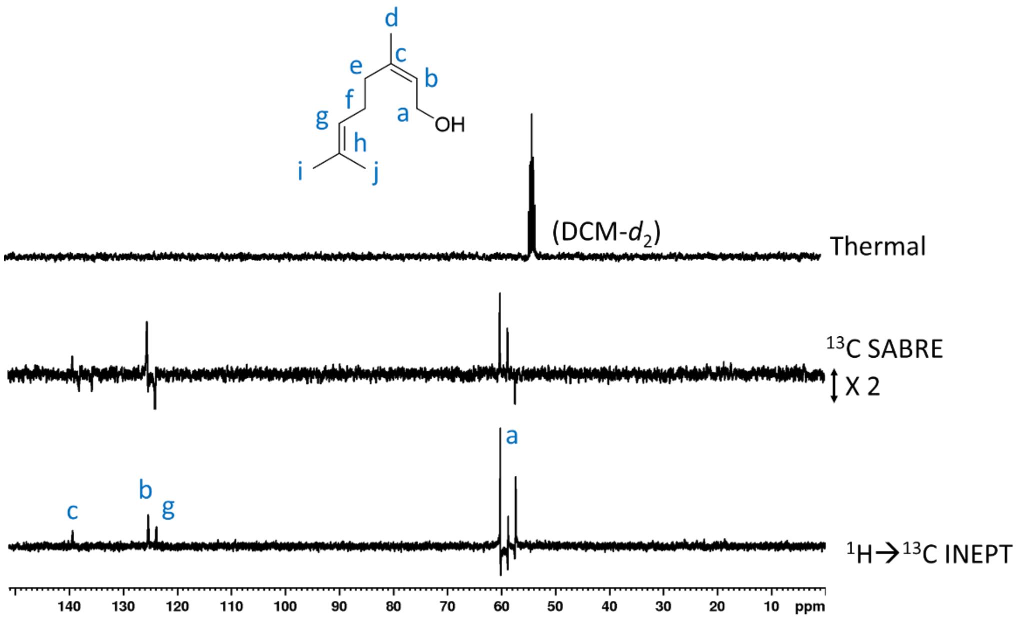

Figure S5: Single scan thermally polarised (above) and SABRE-Relay hyperpolarised (middle and lower) ^{13}C NMR spectra for a sample of [IrCl(COD)(IMes)] (5 mM), NH_3 (25 mM), 2 (25 mM) and pH_2 (3 bar) in DCM-d_2 (0.6 mL). The hyperpolarised spectrum is recorded immediately after shaking the sample for 10 seconds with fresh pH_2 at 6.5 mT. The middle spectrum uses a single 90° pulse for ^{13}C detection whereas the INEPT sequence used in the lower spectrum transfers magnetisation from the ^{1}H domain to the ^{13}C domain via radiofrequency excitation (see

section S1). Note that the lower spectrum is not shown on the same vertical scale as the middle and upper. The associated signal enhancements are given in Table S5.

Table S5: ^1H and ^{13}C NMR signal enhancements for 2 measured from a sample containing [IrCl(COD)(IMes)] (5 mM), NH_3 (25 mM), 2 (25 mM) and pH_2 (3 bar) in DCM-d_2 (0.6 mL). Example spectra used to calculate these enhancements is shown in Figure S4 and S5. ^{13}C NMR signal enhancements are calculated from data recorded with a single 90° detection pulse.

^1H		^{13}C	
Resonance	Enhancement (per proton, fold)	Resonance	Enhancement (per ^{13}C, fold)
OH	71 ± 1	OH	--
a	152 ± 11	a	164
b	21 ± 1	b	128
c	-	c	20
d and j (overlap)	41 ± 3	g	136
e and f (overlap)	37 ± 2		
g	0		
h	-		
i	22 ± 1		
Total	43 ± 3	Total	112

S2.3: SABRE-Relay hyperpolarisation of 3

A sample of **3** (25 mM) was exposed to pH_2 (3 bar) in the presence of NH_3 (25 mM) and the precatalyst [IrCl(COD)IMes] for several hours. The sample was then shaken with pH_2 at 6.5 mT for 10 seconds before spectral acquistion was performed at 9.4 T. Example ^1H and ^{13}C NMR spectra are shown in Figures S6 and S7 respecitvely with the associated NMR signal enhancments being given in Table S6.

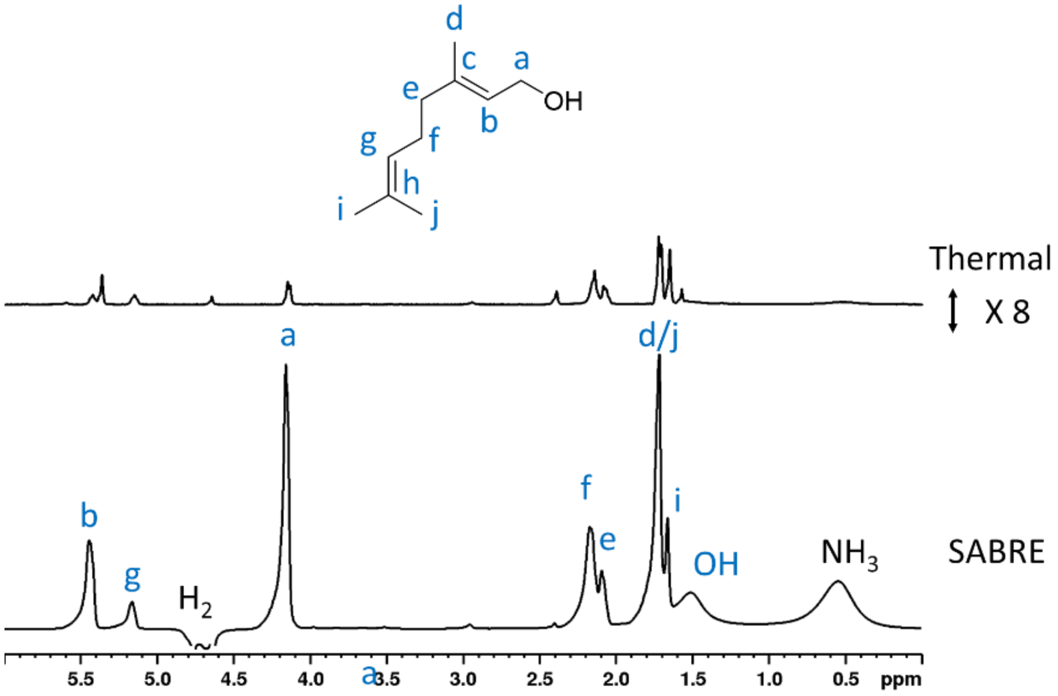

Figure S6: Single scan thermally polarised (above) and SABRE-Relay hyperpolarised (lower) ^1H NMR spectra for a sample of [IrCl(COD)(IMes)] (5 mM), NH_3 (25 mM), 3 (25 mM) and pH_2 (3 bar) in DCM-d_2 (0.6 mL). The hyperpolarised NMR spectrum is recorded immediately after shaking the sample for 10 seconds with fresh pH_2 at 6.5 mT. The associated signal enhancements are given in Table S6.

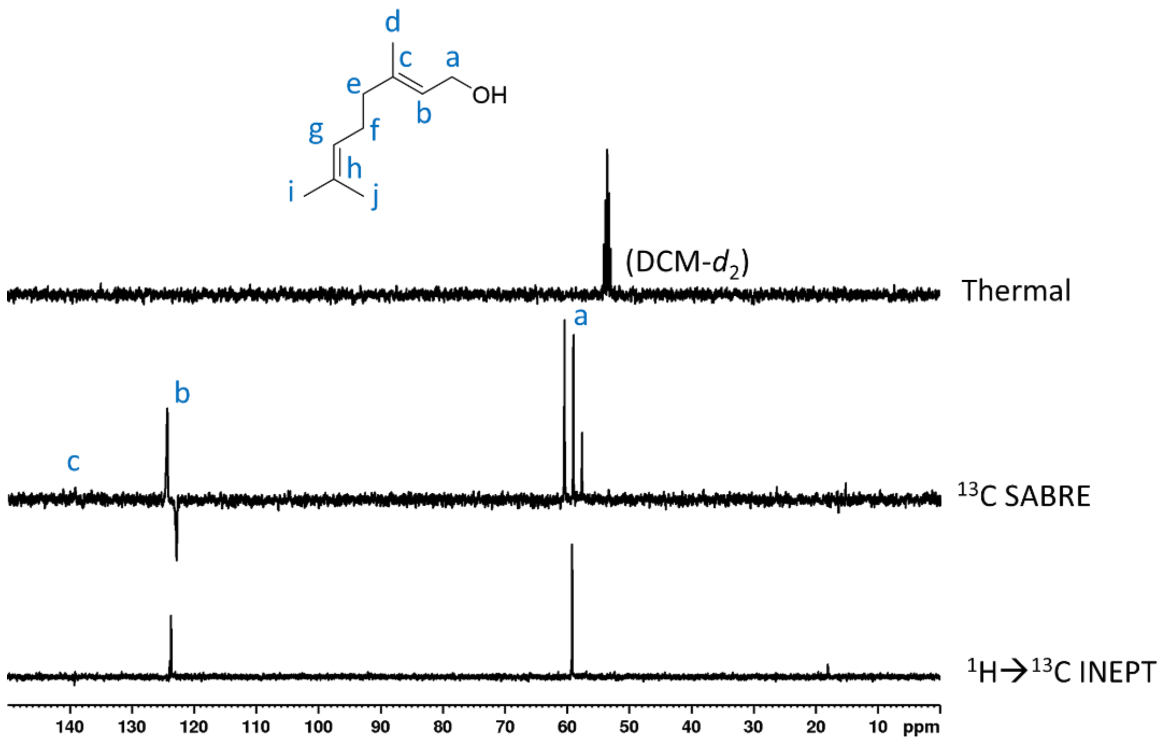

***Figure S7:** Single scan thermally polarised (above) and SABRE-Relay hyperpolarised (middle and lower) ^{13}C NMR spectra for a sample of [IrCl(COD)(IMes)] (5 mM), NH_3 (25 mM), 3 (25 mM) and pH_2 (3 bar) in DCM-d_2 (0.6 mL). The hyperpolarised spectrum is recorded immediately after shaking the sample for 10 seconds with fresh pH_2 at 6.5 mT. The middle spectrum uses a single 90° pulse for ^{13}C detection whereas the INEPT sequence used in the lower spectrum transfers magnetisation from the ^1H domain to the ^{13}C domain via radiofrequency excitation (see section S1). Note that the lower spectrum is not shown on the same vertical scale as the middle and upper. The associated signal enhancements are given in Table S6.*

***Table S6:** ^1H and ^{13}C NMR signal enhancements for 3 measured from a sample containing [IrCl(COD)(IMes)] (5 mM), NH_3 (25 mM), 3 (25 mM) and pH_2 (3 bar) in DCM-d_2 (0.6 mL). Example spectra used to calculate these enhancements is shown in Figure S6 and S7. ^{13}C NMR signal enhancements are calculated from data recorded with a single 90° detection pulse.*

^1H		^{13}C	
Resonance	Enhancement (per proton, fold)	Resonance	Enhancement (per ^{13}C, fold)
OH	70 ± 1	OH	-
a	142 ± 7	a	843
b	49 ± 2	b	834
c	-	c	62
d and j (overlap)	50 ± 4		
e	28 ± 1		
f	46 ± 2		
g	32 ± 1		
h	-		
i	25 ± 1		
Total	108 ± 4	Total	579

S2.4 SABRE-Relay hyperpolarisation of 4

The structure of 4 is shown in Figure S8 and its NMR characterisation data is shown in Table S7. It was hyperpolarised by shaking a sample containing [IrCl(COD)IMes] (5 mM), NH_3 (55 mM), and **4** (25 mM) in dichloromethane-d_2 (0.6 mL) with pH_2 (3 bar) at 6.5 mT for 10 seconds before spectral acquisition was performed at 9.4 T. The pH_2 shaking was performed after the sample has been left to react with H_2 (3 bar) overnight to form $[Ir(H)_2(IMes)(NH_3)_3]Cl$. ^1H and ^{13}C NMR signal enhancements are shown in Table S7 with an example ^{13}C NMR spectra shown in Figure S9 (^1H NMR spectra are shown in Figure 3 of the main paper).

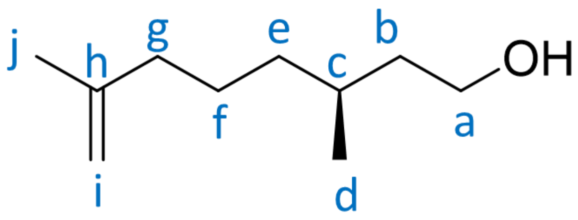

Figure S8: Structure of 4. The NMR data and signal enhancements for the labelled resonances are given in Table S7.

Table S7: NMR characterisation data and signal enhancements for 4 in dichloromethane-d_2 at 298 K. The resonance labels correspond to those shown in Figure S9.

Resonance	^1H (ppm)	^1H Enhancement /fold	^{13}C {^1H} (ppm)	^{13}C Enhancement /fold
OH	3.65	129 ± 6	-	-
a	4.13	148 ± 7	60.1	135
b	1.40	70 ± 5 (overlap with f)	40.1	61 (overlap with e and g)
c	1.71	58 ± 3 (overlap with j)	29.4	0
d	0.94	50 ± 3	19.2	0
e	1.23	33 ± 2	37.4	61 (overlap with b and g)
f	1.37	70 ± 5 (overlap with b)	25.5	0
g	2.06	49 ± 3	38.6	61 (overlap with b and e)
h	-	-	131.1	0
i	5.14, 5.42	46 ± 3, 7 ± 1	124.9	168
j	1.65	58 ± 3 (overlap with c)	17.9	0
Total		47 ± 2		55

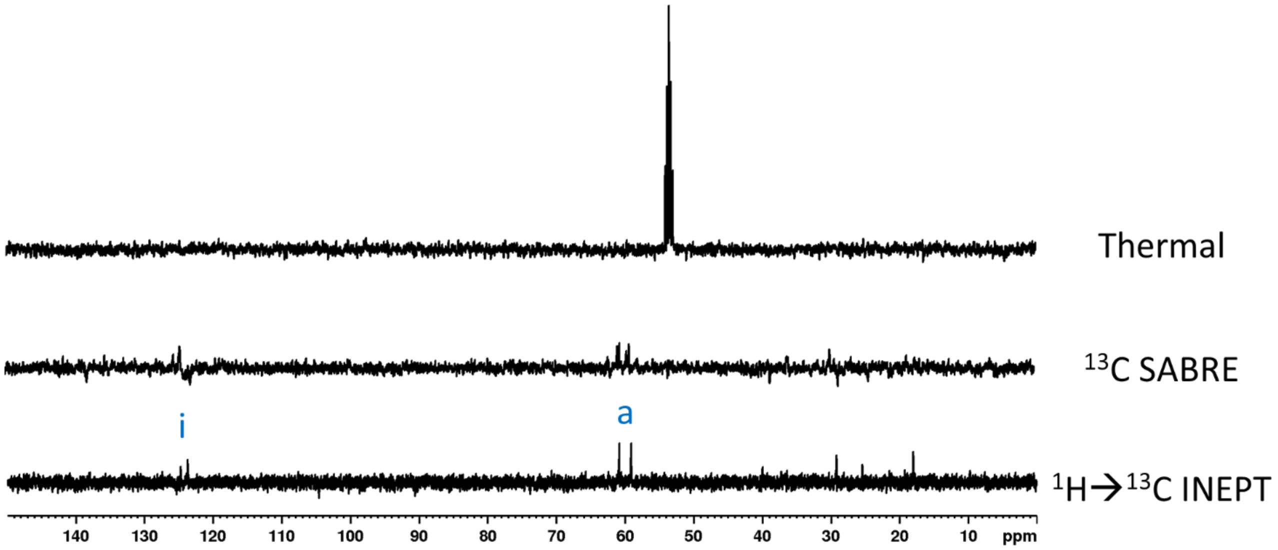

Figure S9: Example single scan thermally polarised (above), direct ^{13}C detection using a single 90° pulse (middle) and $^1H \rightarrow {}^{13}C$ INEPT (lower) for a sample of [IrCl(COD)(IMes)] (5 mM), NH_3 (55 mM), 4 (25 mM) and pH_2 (3 bar) in DCM-d_2 (0.6 mL) following pH_2 shaking for 10 seconds at 6.5 mT. The INEPT spectrum is not shown on the same vertical scale. The associated signal enhancements are given in Table S7.

S2.5 SABRE-Relay hyperpolarisation of 5

The structure of **5** is shown in Figure S10 and its NMR characterisation data is shown in Table S8. It was hyperpolarised by shaking a sample containing [IrCl(COD)IMes] (5 mM), NH_3 (40 mM), and **4** (25 mM) in dichloromethane-d_2 (0.6 mL) with $p$$H_2$ (3 bar) at 6.5 mT for 10 seconds before spectral acquisition was performed at 9.4 T. The $p$$H_2$ shaking was performed after the sample has been left to react with H_2 (3 bar) overnight to form $[Ir(H)_2(IMes)(NH_3)_3]Cl$. ^1H and ^{13}C NMR signal enhancements are also shown in Table S8 with some example NMR spectra shown in Figure S11.

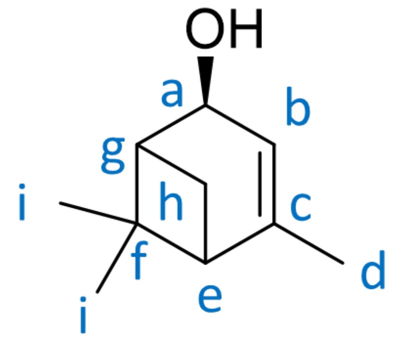

Figure S10: Structure of 5. The NMR data and signal enhancements for the labelled resonances are given in Table S8.

Table S8: NMR characterisation data and signal enhancements for 4 in dichloromethane-d_2 at 298 K. The resonance labels correspond to those shown in Figure S11.

Resonance	^1H (ppm)	^1H Enhancement /fold	^{13}C {^1H} (ppm)	^{13}C Enhancement /fold
a	4.46	302 ± 9	73.4	164 ± 11
b	5.38	38 ± 1	119.7	190 ± 10
c	-	-	147.1	57 ± 18
d	1.77	203 ± 7	18.5	0
e	2.27	207 ± 6	47.6	281 ± 27 (overlap with g)
f	-	-	38.9	205 ± 25 (overlap with h)
g	2.02	220 ± 5	48.2	281 ± 27 (overlap with e)
h	1.32, 2.47	117 ± 6 (overlap with i)	35.5	205 ± 25 (overlap with f)
i	1.09, 1.38	117 ± 6 (overlap with j)	26.3	0
Total		126 ± 6		138 ± 9

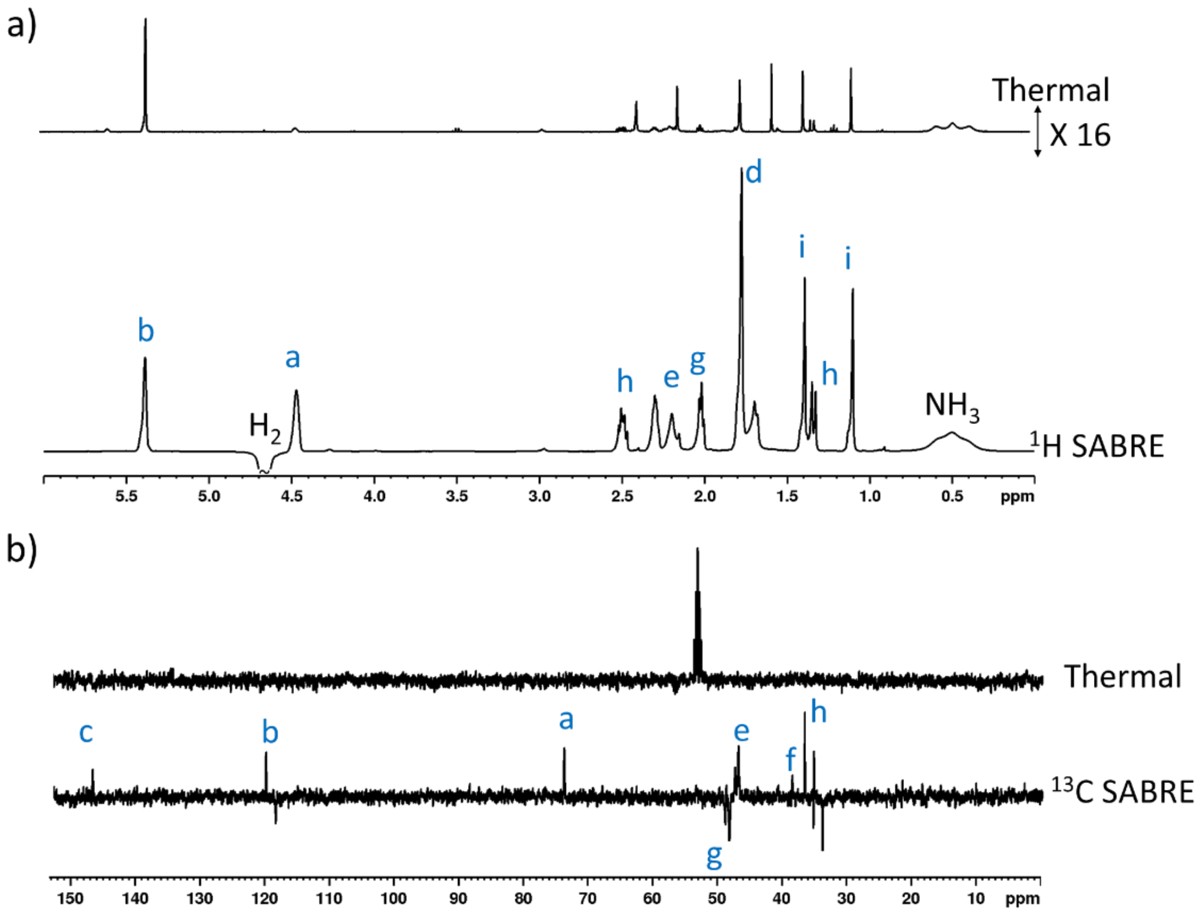

Figure S11: *a) Example single scan thermally polarised (above) and SABRE-Relay hyperpolarised (lower) ^{1}H NMR spectra for a sample of [IrCl(COD)(IMes)] (5 mM), NH_3 (40 mM), 5 (25 mM) and pH_2 (3 bar) in DCM-d_2 (0.6 mL). The hyperpolarised spectra are recorded immediately after shaking the sample for 10 seconds with fresh pH_2 at 6.5 mT. Note that hyperpolarised and thermal spectra are shown on the same vertical scale. b) example single scan thermally polarised (above), direct ^{13}C detection using a single 90° pulse (lower) following fresh pH_2 shaking for the same sample in a). Note a $^{1}H \rightarrow ^{13}C$ INEPT is shown in Figure 3b of the main paper. The associated signal enhancements are given in Table S8.*

S2.6 SABRE-Relay hyperpolarisation of 6

The structure of **6** is shown in Figure S12 and its NMR characterisation data is shown in Table S9. It was hyperpolarised by shaking a sample containing [IrCl(COD)IMes] (5 mM), NH_3 (40 mM), and **6** (25 mM) in dichloromethane-d_2 (0.6 mL) with $p$$H_2$ (3 bar) at 6.5 mT for 10 seconds before spectral acquisition was performed at 9.4 T. The $p$$H_2$ shaking was performed after the sample has been left to react with H_2 (3 bar) overnight to form $[Ir(H)_2(IMes)(NH_3)_3]Cl$. ^{1}H NMR signal enhancements are also shown in Table S9 with example spectra shown in FIugre 3c of the main paper.

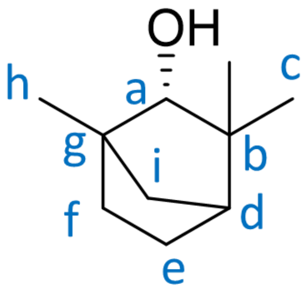

Figure S12: Structure of 6. The NMR data and signal enhancements for the labelled resonances are given in Table S9.

Table S9: NMR characterisation data and signal enhancements for 6 in dichloromethane-d_2 at 298 K. The resonance labels correspond to those shown in Figure S12.

Resonance	^{1}H (ppm)	^{1}H NMR signal enhancement /fold	^{13}C {^{1}H} (ppm)
OH	2.05 (1H)	33 ± 1	-
a	3.29 (1H)	45 ± 4	84.81
b	-	-	39.04
c	0.89 (3H), 1.02 (3H)	5 ± 1	20.23, 30.63
d	1.71 (3H, overlap with e and f)	11 ± 1 (overlap)	48.08
e	1.43 (1H), 1.69 (3H, overlap with d and f)	11 ± 1 (overlap)	26.09
f	1.05 (1H), 1.69 (3H, overlap with d and e)	11 ± 1 (overlap)	25.09
g	-	-	49.10
h	1.12 (3H)	6 ± 1	19.33
i	1.16 (1H), 1.50 (1H)	11 ± 1 (overlap)	41.04
Total		10 ± 1	

S2.7 SABRE-Relay hyperpolarisation of 7

The structure of **7** is shown in Figure S13 and its NMR characterisation data is shown in Table S10. It was hyperpolarised by shaking a sample containing [IrCl(COD)IMes] (5 mM), NH_3 (30 mM), and **7** (25 mM) in dichloromethane-d_2 (0.6 mL) with pH_2 (3 bar) at 6.5 mT for 10 seconds before spectral acquisition was performed at 9.4 T. The pH_2 shaking was performed after the sample has been left to react with H_2 (3 bar) overnight to form $[Ir(H)_2(IMes)(NH_3)_3]Cl$. ^1H and ^{13}C NMR signal enhancements are also shown in Table S10 with some example ^{13}C NMR spectra shown in Figure S14. Note that ^1H NMR spectra are shown in Figure 3d of the main paper.

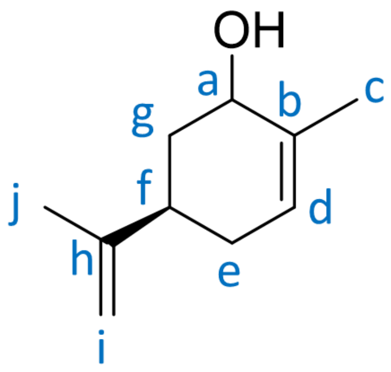

Figure S13: Structure of 7. The NMR data and signal enhancements for the labelled resonances are given in Table S10.

Table S10: NMR characterisation data and signal enhancements for 7 in dichloromethane-d_2 at 298 K. The resonance labels correspond to those shown in Figure S13. Values for one of the diastereomers of 7 are shown in black, with the other being given in red.

Resonance	^1H (ppm)	^1H Enhancement /fold	^{13}C {^1H} (ppm)	^{13}C Enhancement /fold
OH	2.91 (br) 2.91 (br)	21 ± 1 (average for both)	-	-
a	4.02 4.21	70 ± 4 66 ± 4	68.13 70.58	139 ± 8 (average for both)
b	-	-	134.56 136.76	59 ± 3 (average for both)
c	1.82 1.77 or 1.78 (overlap with j)	27 ± 3 (average for both, overlap with j)	20.68 or 20.71 (overlap with j) 18.85	0
d	5.60 5.52	42 ± 4 41 ± 3	124.87 123.41	44 ± 6 (average for both)
e	2.16 2.09	36 ± 2 (average for both)	35.24 40.86	215 ± 18 (average for both) (overlap with f and g)
f	2.39 2.30	30 ± 3 (average for both	31.07/31.15 31.07/31.15	215 ± 18 (average for both) (overlap with e and g)
g	1.62 1.52	30 ± 2 35 ± 3	36.86 38.13	215 ± 18 (average for both) (overlap with e and f)
h	-	-	149.20/149.50 149.20/149.50	26 ± 2 (average for both)
i	4.77/4.78 4.77/4.78	18 ± 1 (average for both	108.72/108.83 108.72/108.83	0
j	1.77 or 1.78 (overlap with c) 1.77 or 1.78 (overlap with c)	27 ± 3 (average for both, overlap with c)	20.71 or 20.68 (overlap with c) 20.71 or 20.68 (overlap with c)	0
Total		32 ± 3 (average for both)		92 ± 7 (average for both)

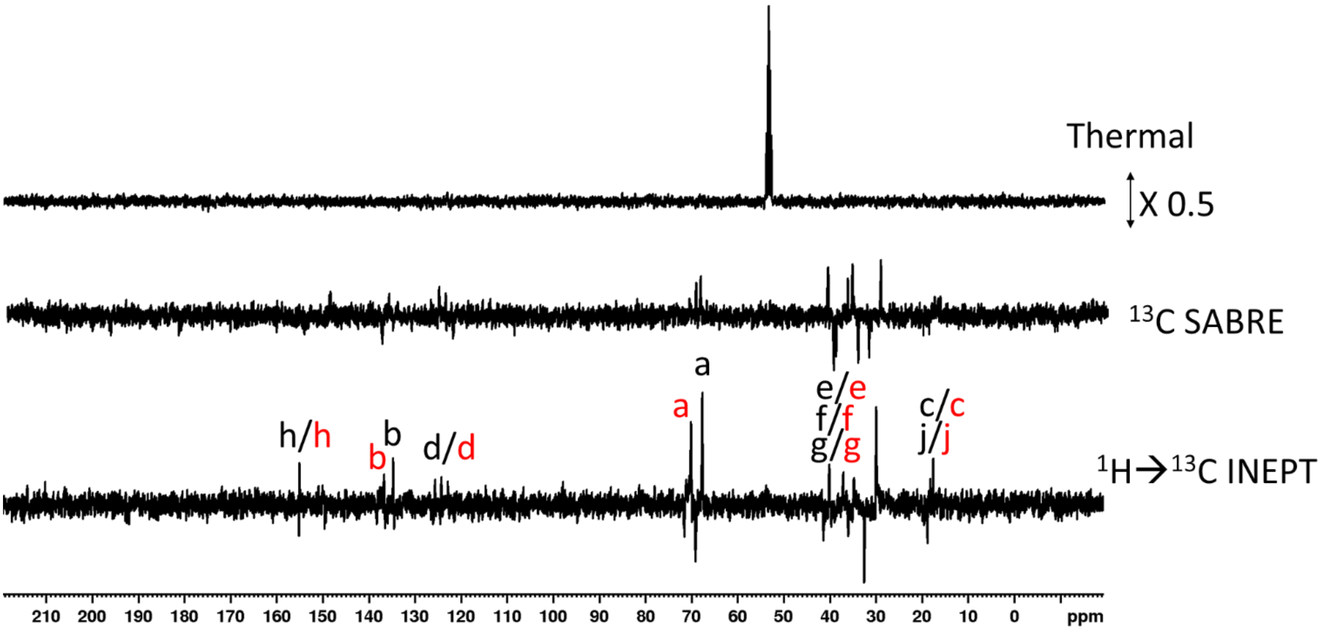

***Figure S14:** Example single scan thermally polarised (above), and SABRE-Relay hyperpolarised direct ^{13}C detection using a single 90° pulse (middle) and SABRE-Relay hyperpolarised $^{1}H \rightarrow ^{13}C$ INEPT (lower) (lower) NMR spectra for a sample of [IrCl(COD)(IMes)] (5 mM), NH_3 (30 mM), 7 (25 mM) and pH_2 (3 bar) in DCM-d_2 (0.6 mL). The hyperpolarised spectra are recorded immediately after shaking the sample for 10 seconds with fresh pH_2 at 6.5 mT. The INEPT spectrum is not shown on the same vertical scale. The associated signal enhancements are given in Table S10.*

S2.8 SABRE-Relay hyperpolarisation of 8

The structure of **8** is shown in Figure S15 and its NMR characterisation data is shown in Table S11. It was hyperpolarised by shaking a sample containing [IrCl(COD)IMes] (5 mM), NH_3 (35 mM), and **8** (25 mM) in dichloromethane-d_2 (0.6 mL) with pH_2 (3 bar) at 6.5 mT for 10 seconds before spectral acquisition was performed at 9.4 T. The pH_2 shaking was performed after the sample has been left to react with H_2 (3 bar) overnight to form $[Ir(H)_2(IMes)(NH_3)_3]Cl$. ^1H and ^{13}C NMR signal enhancements are also shown in Table S11 with some example spectra shown in Figure S16.

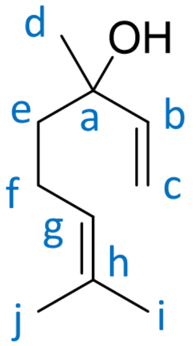

Figure S15: Structure of 8. The NMR data and signal enhancements for the labelled resonances are given in Table S11.

Table S11: NMR characterisation data and signal enhancements for 8 in dichloromethane-d_2 at 298 K. The resonance labels correspond to those shown in Figure S15.

Resonance	^1H (ppm)	^1H Enhancement /fold
a	-	-
b	5.96	7 ± 1
c	5.16 (overlap with g)	2 ± 1 (overlap with g)
d	1.29	62 ± 10
e	2.05 (overlap with f)	61 ± 5 (overlap with f)
f	2.05 (overlap with e)	61 ± 5 (overlap with e)
g	5.16 (overlap with c)	2 ± 1 (overlap with c)
h	-	-
i	1.72	0
j	1.64	0
Total		38 ± 4

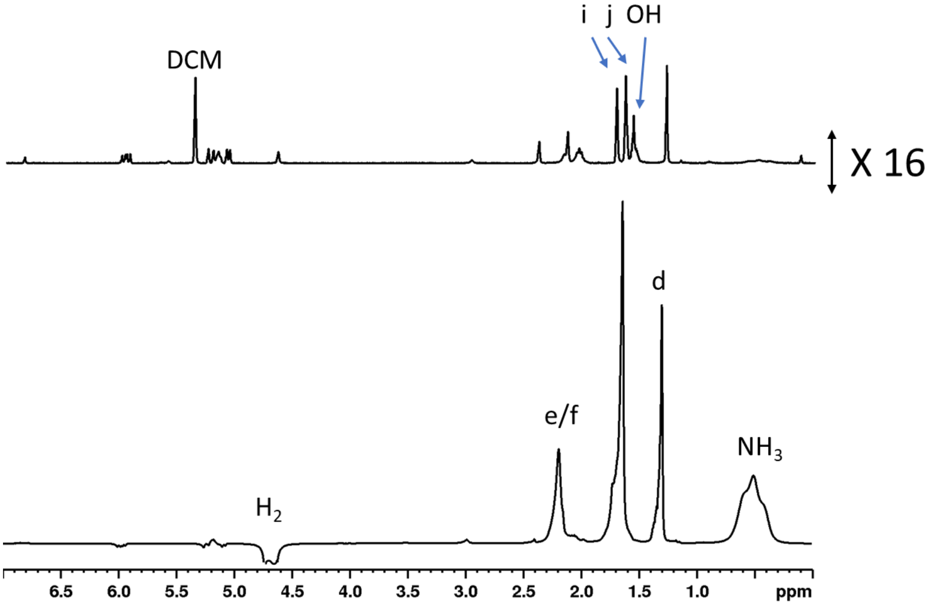

Figure S16: Example single scan thermally polarised (above) and SABRE-Relay hyperpolarised (lower) ^1H NMR spectra for a sample of [IrCl(COD)(IMes)] (5 mM), NH_3 (35 mM), 8 (25 mM) and pH_2 (3 bar) in DCM-d_2 (0.6 mL). The hyperpolarised spectra are recorded immediately after shaking the sample for 10 seconds with fresh pH_2 at 6.5 mT.

S3: Optimising SABRE-Relay hyperpolarisation

S3: Effect of carrier on SABRE-Relay hyperpolarisation of 1

Samples of **1** (25 mM) were exposed to *p*H_2 (3 bar) in the presence of the indicated carrier molecule (25 mM) and the precatalyst [IrCl(COD)IMes] for several hours. The samples were then shaken with *p*H_2 at 6.5 mT for 10 seconds before spectral acquistion was performed at 9.4 T. Example ^{1}H NMR spectra are shown in Figure S17, with the associated NMR signal enhancments given in Table S12.

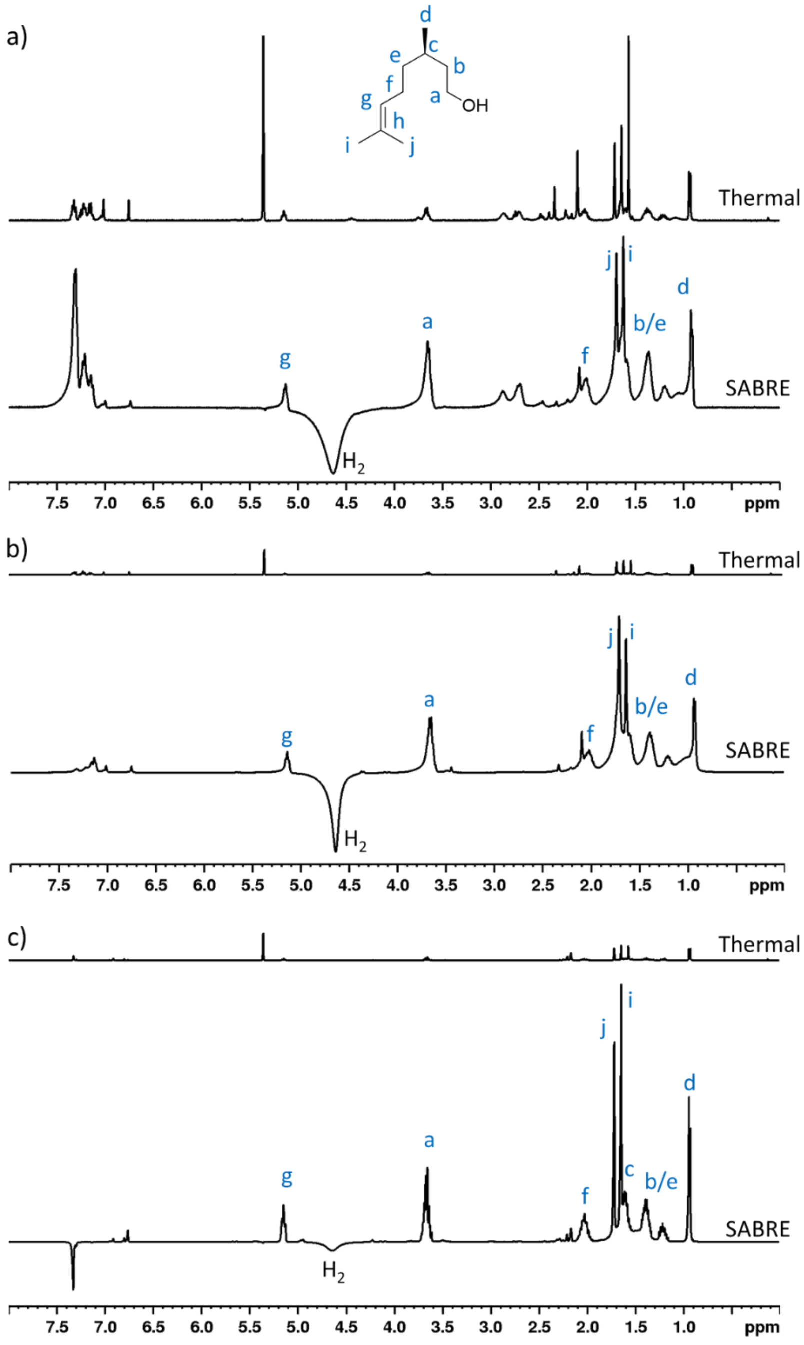

Figure S17: Single scan thermally polarised (above) and SABRE-Relay hyperpolarised (lower) ^{1}H NMR spectra for a sample of [IrCl(COD)(IMes)] (5 mM), 1 (25 mM) and pH_2 (3 bar) in DCM-d_2 (0.6 mL) with the carriers a) phenethylamine b) phenethylamine-d_4 c) benzylamine-d_7. The hyperpolarised spectra are recorded immediately after shaking the sample for 10 seconds with fresh pH_2 at 6.5 mT. Note that hyperpolarised and thermal spectra are shown on the same vertical scale, but a) b) and c) are not shown on the same vertical scale. The associated signal enhancements are given in Table S12.

Table S12: ^{1}H NMR signal enhancements for 1 measured from samples containing [IrCl(COD)(IMes)] (5 mM), carrier (25 mM), 1 (25 mM) and pH$_2$ (3 bar) in DCM-d$_2$ (0.6 mL). Example spectra used to calculate these enhancements are shown in Figure S17.

	Phenethylamine	Phenethylamine-d_4	Benzylamine-d_7
Resonance	Enhancement (per proton, fold)	Enhancement (per proton, fold)	Enhancement (per proton, fold)
a	6 ± 1	39 ± 1	32 ± 2
b	6 ± 1 (overlap)	27 ± 1 (overlap)	18 ± 1 (overlap)
c	&	&	&
d	4 ± 1	20 ± 1	16 ± 1
e	6 ± 1 (overlap)	27 ± 1 (overlap)	18 ± 1 (overlap)
f	0	15 ± 1	15 ± 1
g	3 ± 1	22 ± 1	26 ± 1
i	3 ± 1	16 ± 2	22 ± 2
j	5 ± 1	28 ± 1	23 ± 1
Total	5 ± 1	23 ± 1	20 ± 1

[&]In hyperpolarised spectra the signal for c is hidden under those of i and j. In thermal spectra it overlaps with the cyclooctane signal.

S3.2: Effect of carrier on SABRE-Relay hyperpolarisation of 2

Samples of **2** (25 mM) were exposed to *p*H$_2$ (3 bar) in the presence of the indicated carrier molecule (25 mM) and the precatalyst [IrCl(COD)IMes] for several hours. The sample was then shaken with *p*H$_2$ at 6.5 mT for 10 seconds before spectral acquistion was performed at 9.4 T. ^{1}H NMR signal enhancments for **2** achieved using the different carriers are shown in Table S13.

Table S13: ^{1}H NMR signal enhancements for 2 measured from samples containing [IrCl(COD)(IMes)] (5 mM), carrier (25 mM), 2 (25 mM) and pH$_2$ (3 bar) in DCM-d$_2$ (0.6 mL).

	Phenethylamine	Phenethylamine-d_4	Benzylamine-d_7
Resonance	Enhancement (per proton, fold)	Enhancement (per proton, fold)	Enhancement (per proton, fold)
OH	16 ± 1	13 ± 3	29 ± 5
a	4 ± 1	55 ± 19	79 ± 5
b	0	9 ± 3	18 ± 1
c	-	-	-
d and j (overlap)	4 ± 1	26 ± 6	29 ± 3
e and f (overlap)	4 ± 1	6 ± 1	8 ± 1
g	6 ± 1	7 ± 3	4 ± 1
h	-	-	-
i	5 ± 1	19 ± 6	25 ± 5
Total	6 ± 1	16 ± 4	24 ± 3

S3.2: Effect of carrier on SABRE-Relay hyperpolarisation of 3

Samples of **3** (25 mM) were exposed to *p*H_2 (3 bar) in the presence of the indicated carrier molecule (25 mM) and the precatalyst [IrCl(COD)IMes] for several hours. The sample was then shaken with *p*H_2 at 6.5 mT for 10 seconds before spectral acquistion was performed at 9.4 T. ^{1}H NMR signal enhancments for **3** achieved using the different carriers are shown in Table S14.

Table S14: ^{1}H NMR signal enhancements for 3 measured from samples containing [IrCl(COD)(IMes)] (5 mM), carrier (25 mM), 3 (25 mM) and pH$_2$ (3 bar) in DCM-d$_2$ (0.6 mL).

	Phenethylamine-*d*$_4$	Benzylamine-*d*$_7$
Resonance	Enhancement (per proton, fold)	Enhancement (per proton, fold)
OH	27 ± 2	83 ± 6
a	85 ± 3	142 ± 12
b	15 ± 1	28 ± 3
c	-	-
d and j (overlap)	42 ± 1	27 ± 13
e	15 ± 1	38 ± 1
f	26 ± 1	14 ± 3
g	21 ± 1	28 ± 5
h	-	-
i	25 ± 1	84 ± 5
Total	84 ± 1	113 ± 9

S3.4 Effect of NH_3 concentration on SABRE-Relay hyperpolarisation of 1

Samples of **1** (30 mM) were exposed to *p*H_2 (3 bar) in the presence of NH_3 and the precatalyst [IrCl(COD)IMes] for several hours. The sample was then shaken with *p*H_2 at 6.5 mT for 10 seconds before spectral acquistion was performed at 9.4 T. The amine concentration was then lowered in the sample by bubbling N_2 gas through the solution under an inert atmosphere. The sample voluem was returned to 0.6 mL if necessary and degassed agin to remove dissolved oxygen. This lowered the NH_3 amount from 60 mM to 30 mM ^{1}H and ^{13}C NMR signals enhancements at each NH_3 loading were recorded and the results are displayed in Figure S18.

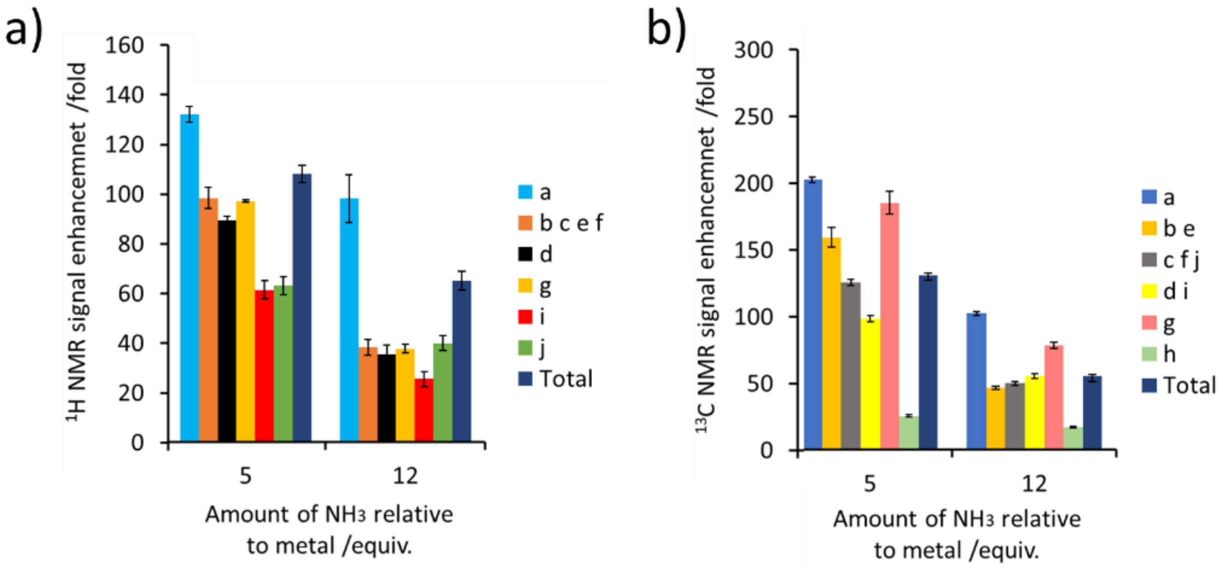

Figure S18: Comparison of a) ^{1}H and b) ^{13}C NMR signal enhancements for 1 when a sample of it (25 mM) is shaken with [IrCl(COD)(IMes)] (5 mM), NH$_3$ and pH$_2$ (3 bar) in DCM-d$_2$ (0.6 mL) for 10 seconds at 6.5 mT.

S4: Hyperpolarisation of rose geranium oil

S4.1 Characterisation of rose geranium oil

The rose geranium oil studied was purchased from Nikura (https://nikura.com/products/rose-geranium-essential-oil) and detailed information regarding its composition is available from their website (under documents tab). The components of the rose geranium oil according to the manufacturer are: **1** (21.1%), **3** (17.7%), **7** (10.2%), isomenthone (8.1%), citronellyl formate (7.9%), geranyl formate (5.9%), menthone (1.6%), geranyl propionate (1.5%), **2** (1%), citronellyl tiglate (1%), alpha-terpineol (1%), geranyl butyrate (1%), cis-rose oxide (1%), citronellyl butyrate (0.8%), beta-caryophyllene (0.8%), myrcene (0.8%), geranyl isobutyrate (0.6%), alpha-pinene (0.6%), geranyl acetate (0.6%), beta-pinene (0.5%), trans-rose oxide (0.5%), geranial (0.4%), l-limonene (0.5%), trans beta-ocimene (0.3%), cis beta-ocimene (0.2%), neryl formate (0.1%), p-cymene (0.1%), neral (0.1%), terpinolene (0.1%), alpha phellandrene (0.1%).

We performed an NMR analysis of a concentrated sample of the rose geranium oil (300 µL in dichloromethane-d_2 300 µL) for reference. Example ^{1}H and ^{13}C NMR spectra are shown in Figures S19-S20 with key resonances assigned (assignments were supported by 2D NMR experiments, not shown).

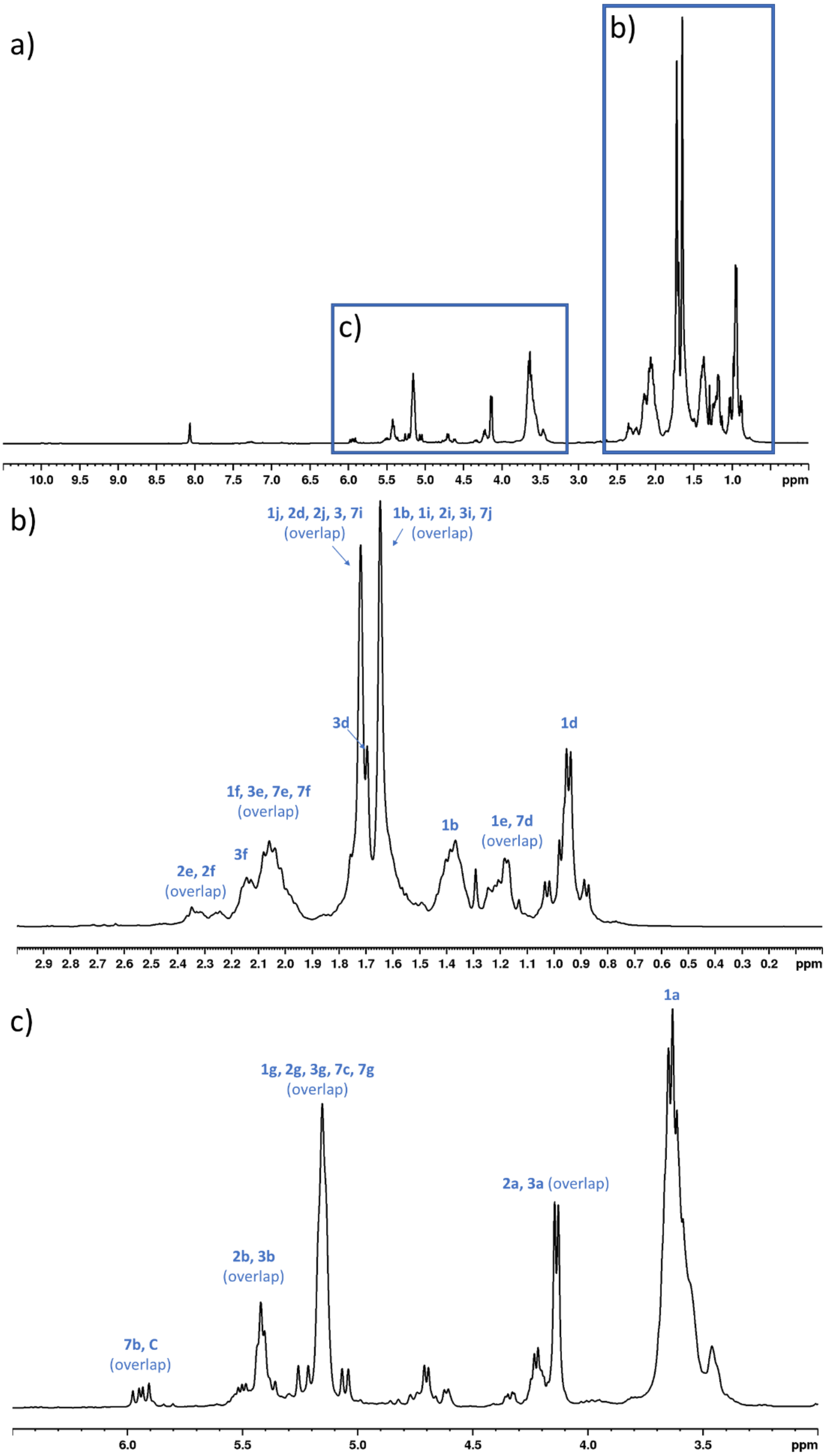

Figure S19: a) ^{1}H NMR spectrum of rose geranium oil (0.3 mL) in DCM-d_2 (0.3 mL) with zoomed in regions shown in b) and c). Spectra are labelled for the major components 1, 3 and 7, with signals for 2 also labelled (which overlap with those of 3) according to the labelling system used in Figure 1c of the main paper, and section S2 of the supporting information. Other resonances for major components such as isomenthone (A), citronellyl formate (B), and geranyl formate (C) are also labelled.

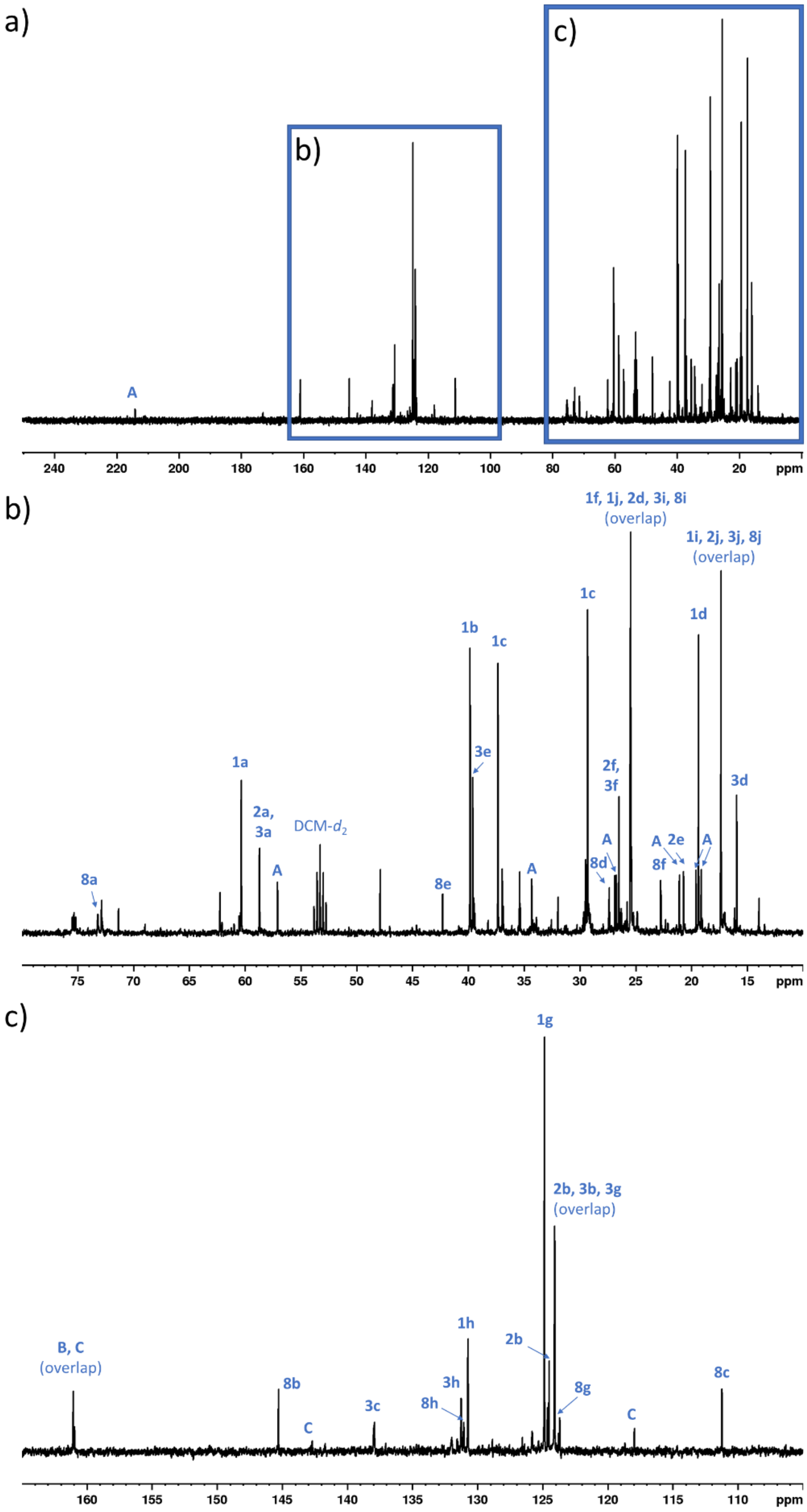

***Figure S20:** a) ^{13}C NMR spectrum of rose geranium oil (0.3 mL) in DCM-d_2 (0.3 mL) with zoomed in regions shown in b) and c). Spectra are labelled for the major components 1, 3 and 7, with signals for 2 also labelled (which overlap with those of 3) according to the labelling system used in Figure 1c of the main paper, and section S2 of the supporting information. Other resonances for major components such as isomenthone (A), citronellyl formate (B), and geranyl formate (C) are also labelled.*

S4.2 Hyperpolarisation experiments

$[Ir(H)_2(IMes)(NH_3)_3]Cl$ was formed *in situ* by reaction of [IrCl(COD)IMes] (5 mM) and NH_3 (25 mM), in dichloromethane-d_2 (0.6 mL) with H_2 (3 bar) overnight at room temperature. At this point it was shaken with pH$_2$ (3 bar) at 6.5 mT for 10 seconds to record a control spectrum without any rose geranium oil present. An addition of 7.17 µL of rose geranium oil was made to the NMR tube inside a glovebox as a precaution to limit moisture from the air entering the tube. For more dilute rose oil concentrations, 0.5 µL of this pure rose geranium oil was diluted in 100 µL dichloromethane-d_2. Then, either 2 µL, 5µL, or 30 µL of this diluted solution were added to produce the indicated **1** or **3** concentrations (40, 92 and 554 µM for **1** and 29, 72 and 431 µM for **3**). These concentrations were determined by spiking a stock solution of rose geranium oil with a known amount of tetramethylsilane and comparing the integral intensity of the internal standard to **1** and **3**.
The signal enhancements recorded for **1** and **3** in these mixtures are shown in Table S15.

Table S15: ^{1}H NMR signal enhancements recorded when samples of rose geranium oil are hyperpolarised using SABRE-Relay. Note the resonance labels are according to the scheme depicted in Figures S1 and S3. Note this table only shows the distinct resonances arising from components of the rose geranium oil.

^{1}H (ppm)	Component of the rose oil	Resonance	^{1}H Enhancement /fold **1**: 26.5 mM **3**: 20.6 mM	^{1}H Enhancement /fold **1**: 554 µM **3**: 431 µM
5.43	**3**	b	25 ± 2	127 ± 5
5.16	**1** and **3**	g	12 ± 1	35 ± 4
4.13	**3**	a	33 ± 3	75 ± 2
3.67	**1**	a	36 ± 3	98 ± 4
1.38	**1**	b and e	13 ± 1	40 ± 5
1.19	**1**	e	6 ± 1	19 ± 2
0.94	**1**	d	10 ± 1	42 ± 4
